# Buty and the beast: the complex role of butyrate in Parkinson’s disease

**DOI:** 10.3389/fphar.2024.1388401

**Published:** 2024-04-17

**Authors:** Joshua D. Elford, Nanette Becht, Johan Garssen, Aletta D. Kraneveld, Paula Perez-Pardo

**Affiliations:** ^1^ Division of Pharmacology, Utrecht Institute for Pharmaceutical Sciences, Faculty of Science, Utrecht University, Utrecht, Netherlands; ^2^ Danone Nutricia Research, Utrecht, Netherlands; ^3^ Department of Neuroscience, Faculty of Science, Vrije Universiteit, Amsterdam, Netherlands

**Keywords:** Parkinson’s disease, short-chain fatty acid, butyrate, gut-brain axis, microbiome

## Abstract

Parkinson’s disease (PD) is a complex neurodegenerative disease which is often associated with gastrointestinal (GI) dysfunction. The GI tract is home to a wide range of microorganisms, among which bacteria, that can influence the host through various mechanisms. Products produced by these bacteria can act in the gut but can also exert effects in the brain via what is now well established to be the microbiota-gut-brain axis. In those with PD the gut-bacteria composition is often found to be different to that of non-PD individuals. In addition to compositional changes, the metabolic activity of the gut-microbiota is also changed in PD. Specifically, it is often reported that key producers of short chain fatty acids (SCFAs) as well as the concentration of SCFAs themselves are altered in the stool and blood of those with PD. These SCFAs, among which butyrate, are essential nutrients for the host and are a major energy source for epithelial cells of the GI tract. Additionally, butyrate plays a key role in regulating various host responses particularly in relation to inflammation. Studies have demonstrated that a reduction in butyrate levels can have a critical role in the onset and progression of PD. Furthermore, it has been shown that restoring butyrate levels in those with PD through methods such as probiotics, prebiotics, sodium butyrate supplementation, and fecal transplantation can have a beneficial effect on both motor and non-motor outcomes of the disease. This review presents an overview of evidence for the altered gut-bacteria composition and corresponding metabolite production in those with PD, with a particular focus on the SCFA butyrate. In addition to presenting current studies regarding SCFA in clinical and preclinical reports, evidence for the possibility to target butyrate production using microbiome based approaches in a therapeutic context is discussed.

## 1 Introduction

Parkinson’s disease (PD) is a progressive neurodegenerative disorder with an increasing prevalence, particularly in industrialized countries, with as many as 0.3% of the population estimated to have PD (Hayes, 2019). This proportion, however, greatly increases with age with as many as 3% of those above the age of 80 having PD (Dexter and Jenner, 2013; Hayes, 2019). The misfolding of the PD-associated hallmark protein α-Synuclein (αSyn) leads to its aggregation and subsequent inclusion in Lewy bodies within the cytoplasm of cells of the midbrain and cortex (Hayes, 2019). These Lewy bodies are found within the neurons and are typically associated with the death of dopaminergic (DA) neurons (Kouli et al., 2018). The substantia nigra (SN) has a high concentration of DA neurons and is mainly involved in motor control. Loss of these DA neurons in the SN is therefore directly correlated to the severity of motor symptoms in PD (Kouli et al., 2018).

In addition to motor symptoms, PD also displays an array of non-motor symptoms (Lee and Koh, 2015) which have been seen to precede motor symptom onset by, in some cases, several years (Hayes, 2019). Prodromal symptoms can include sleep disorders, urinary dysfunction, depression, and sensory abnormalities (e.g., hyposmia), as well as gastrointestinal (GI) dysfunction (e.g., constipation, sialorrhea) (Jankovic, 2008; Kouli et al., 2018). The most common GI symptom, constipation, has been observed to precede diagnosis of PD by as many as 20 years (Chiang and Lin, 2019). Dysfunction of the gastrointestinal (GI) system is often related with a change in microbiota composition. In addition, several studies also have found correlations between disease severity and an altered microbiota (Barichella et al., 2019; Romano et al., 2021; Boertien et al., 2022; Nuzum et al., 2023) (reviewed (Boertien et al., 2019; Bullich et al., 2019; Nuzum et al., 2020; Papić et al., 2022)).

## 2 Gut-brain axis in PD

The gut microbiome encapsulates the variety of fungi, viruses, archaea, and bacteria that colonize the GI tract (Ghaisas et al., 2016). It is implicated in both health and disease, with a number of neurological disorders already being associated with alterations in microbiota composition (Ghaisas et al., 2016; Pellegrini et al., 2018; Cryan et al., 2020; Markidi et al., 2024). There is a two-way communication between the brain and the gut whereby, to name one example, neuronal signals are sent from the brain to the GI tract and the gut can affect the brain via hormone production and uptake of nutrients (Margolis et al., 2021). The microbiota also plays an important part in this communication by, for example, producing different products (e.g., amino acids and short chain fatty acids (SCFAs)), direct interaction with nerve and immune cells of the GI tract, and regulation of hormones (summarized (Cryan et al., 2020)).

In the context of PD, the involvement of the vagus nerve, a key link between the gut and the brain, is suspected due to the observation of αSyn aggregates spreading through the brain in a staged, retrograde manner, seeming to originate from the dorsal motor nucleus of the vagus nerve (in addition to the olfactory tract) (Braak et al., 2003; Hawkes et al., 2007). This spread coupled with αSyn aggregates being observed in the enteric nervous system (ENS) (Bloch et al., 2006; Braak et al., 2006) led Braak and others to propose that the gut, in addition to the nasal tract, can be the site for a neurotropic pathogen to disturb αSyn folding followed by a prion-like transport via the vagus nerve to the brain eventually leading to the development of PD (Hawkes et al., 2007; Rietdijk et al., 2017). Furthermore, preclinical studies have investigated the transport of αSyn along the vagus nerve and visualized its spread in a time dependent manner (Holmqvist et al., 2014).

Further evidence for the vagal route of αSyn transmission is found in patients that have undergone full truncal vagotomy, where complete resection of the vagus nerve is protective for PD development (Svensson et al., 2015). Interestingly, when only partial resection (selective vagotomy) was performed no protective effect was observed after controlling for factors such as age and sex (Svensson et al., 2015). This finding is further supported by preclinical studies whereby hemivagotomy was able to prevent the loss of dopaminergic neurons in the SN in an oral toxin model of PD (Pan-Montojo et al., 2012; Rietdijk et al., 2017). Why hemivagotomy is sufficient to prevent PD onset in an animal model of PD but not in humans is not known, however is likely due to the complex etiology seen in human cases of PD.

Recently, observations of Borghammer and others expand on the findings of Braak et al. and suggest two potential models for the spread of αSyn aggregates with a subtype of patients where the spread appears to originate from the GI tract (so called “body first” subtype) and others where αSyn aggregates appearing to originate in the brain (a “brain first” subtype) (Borghammer and Van Den Berge, 2019). This study used a multimodal imaging battery to cluster *de novo* and prodromal PD cases into these two distinct subtypes. Interestingly, certain symptoms, such as rapid eye movement (REM) sleep behavior disorder and GI related parameters, are closely linked with a “body first” subtype whilst being rarely associated with “brain first” subjects (Borghammer and Van Den Berge, 2019). These subtypes are supported by *in vitro, in vivo,* and clinical evidence however the exact causes and mechanisms that underly PD remain to be elucidated (Rietdijk et al., 2017; Borghammer and Van Den Berge, 2019; Nuzum et al., 2020; Cui et al., 2023).

Involvement of the microbiota gut-brain axis in PD may therefore be especially relevant for (at least) a subset of people with PD. The exact mechanisms involved between the microbiota and the host are multifaceted and SCFAs are a prime example of how microbiota can produce metabolites that directly influence GI tract function and have wider effects. One such example is the ability of SCFAs, and especially butyrate, to modulate inflammatory responses and thereby influence αSyn accumulation and propagation (Kakoty et al., 2021; Kim et al., 2022). SCFAs are primarily a product of the fermentation of non-digestible fibers by gut bacteria (Blaak et al., 2020). Colonocytes are responsible for the uptake of SCFA from the lumen and begin to metabolize the SCFAs for their own energy needs, the unused portion of SCFAs is then transported across the basolateral membrane (den Besten et al., 2013; Blaak et al., 2020; Aho et al., 2021). In PD, among broader changes in the microbiota composition, there have been various reports describing a decrease in SCFA producing species (Bullich et al., 2019; Romano et al., 2021; Nuzum et al., 2023). Studies that examine levels of SCFAs themselves are less abundant, but the few available also describe a similar reduction of SCFA in the GI tract, particularly regarding butyrate (Jackson et al., 2019; Aho et al., 2021). This reduction in intestinal butyrate may have a detrimental outcome for colonocytes due to reduced anti-inflammatory and proliferative effects of butyrate as well as the important role of butyrate in regulating intestinal barrier permeability (Liu et al., 2018; Karunaratne et al., 2020). A reduction in epithelial tight junction (TJ) protein expression and therefore impaired barrier integrity, e.g., leaky gut (Clairembault et al., 2015; Perez-Pardo et al., 2019) is an important feature of PD-GI related symptoms and therefore the ability of butyrate to modulate this presents an interesting opportunity for intervention.

This review aims to explore the role of butyrate in PD. Firstly, the role of butyrate in human health is described. Secondly, a synopsis of research comparing the gut bacteria composition in PD subjects and controls is explored. In addition, recent findings regarding butyrate in PD in both clinical and preclinical studies are summarized. Finally, opportunities to exploit butyrate related pathways are examined and the current potential treatments targeting the disturbed butyrate concentrations in PD are explored, including pre-, pro-, and postbiotics as well as fecal microbiota transplantation (FMT).

## 3 Butyrate in gut health and disease

### 3.1 Microbiota is unique and can be influenced by many factors

The human body plays host to trillions of microbes, with a large proportion found in the GI tract (Ghaisas et al., 2016). Although the absolute number of bacterial cells is similar to that of body cells (estimates place this at 1.3:1 bacterial:human), it has been estimated that the number of unique genes found in the microbiota is as much as 150 times that of the human genome (Ghaisas et al., 2016; Gilbert et al., 2018). The microbial community is composed of many different bacterial species, the exact composition of which is unique for every individual and this remains relatively stable for most of their life (Cresci and Bawden, 2015; Walker and Hoyles, 2023). Various factors influence an individuals’ microbiota composition including: age, health status, ethnicity, geographical location, and diet among others (Cresci and Bawden, 2015; Ghaisas et al., 2016; Pellegrini et al., 2018). Diet and pharmaceutical products are clear front runners in their capacity to modify the composition of an individual’s microbiota (Turnbaugh et al., 2009; Cresci and Bawden, 2015; Schmidt et al., 2018). Intuitively, antibiotics have the largest effect on microbiota composition when compared to other pharmaceutical treatments (Cresci and Bawden, 2015; Schmidt et al., 2018). Such changes caused by antibiotics in microbiome composition are believed to be reversible with time, however some amount of diversity may be lost permanently due to taxa being completely eliminated from the microbiota (Schmidt et al., 2018). Interestingly in relation to PD the use of levodopa, the primary pharmaceutical therapy for PD, has been shown to have interactions with the microbiome. Different bacteria have been shown to possess enzymes that are capable of structural modification to Levodopa including decarboxylation and deamination (van Kessel et al., 2019; 2020). This results in a lowered bioavailability of the treatment and hence a reduction in therapeutic efficacy (van Kessel et al., 2019; 2020) and can also increase the occurrence of side effects due to metabolism products in the GI tract (van Kessel et al., 2020). Furthermore, the use of Levodopa-carbidopa intestinal gel has been shown to influence microbiota composition when compared to Levodopa treatment alone (Melis et al., 2021). What the long term implications of such changes are, remain unclear.

### 3.2 Butyrate is a key source of energy for colonocytes

The production of SCFAs is a key role of the gut bacteria, since these are essential nutrients (Blaak et al., 2020). A majority of the bioavailable SCFAs found in the body originate from the colon, with acetate, propionate, and butyrate being the most abundant (Blaak et al., 2020; Martin-Gallausiaux et al., 2021). Bacteria of the families *Oscillospiraceae* and *Lachnospiraceae* of the Bacillota phylum are the predominant butyrate producers within the human gut (Louis and Flint, 2017; Blaak et al., 2020). Other bacteria can play an important part in the production of butyrate due to the phenomenon of cross-feeding whereby metabolites are exchanged between multiple different species to eventually produce a final product (Rivière et al., 2016; Culp and Goodman, 2023). Thus the actual butyrate production of the bacteria depends on many factors, including pH, diet, and precursor production (Rivière et al., 2016; Martin-Gallausiaux et al., 2021).

Carbohydrates are the main source from which gut bacteria produce butyrate, with the majority being otherwise indigestible fibers (Louis and Flint, 2017). Butyrate is formed by the condensation of two molecules of acetyl-CoA by the enzymes butyrate kinase or butyryl-CoA:acetate CoA-transferase (Louis and Flint, 2017; Blaak et al., 2020). Once produced, the weakly acidic butyrate is present in a deprotonated form in the colon, requiring active transport for absorption (Martin-Gallausiaux et al., 2021). Whilst the undissociated form of butyrate is able to passively diffuse across the colonocyte membrane, this mechanism is of little physiological relevance due to the rapid dissociation of butyrate and proton that occurs in the colon (Astbury and Corfe, 2012; Martin-Gallausiaux et al., 2021).

**FIGURE 1:**
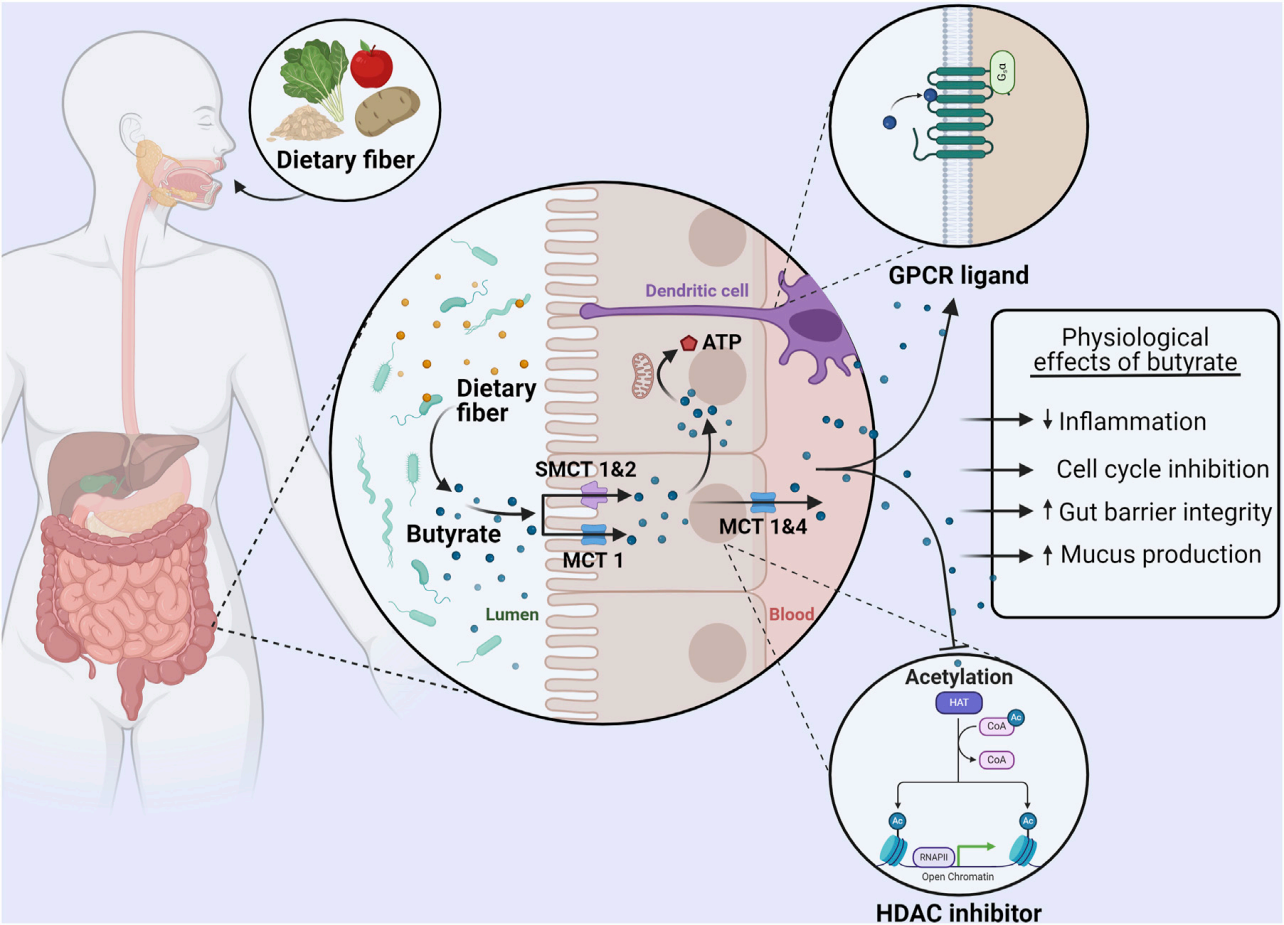
Uptake, metabolism, and physiological effects of butyrate in the colon. Dietary fiber consumption results in the production of butyrate in the gut lumen by the microbiota. Monocarboxylate transporter (MCT) one and sodium-dependent monocarboxylate transporter (SMCT) 1&2 on the apical membrane of colonocytes are responsible for the absorption of butyrate. A majority of butyrate is directly used as an energy source by colonocytes. The remaining butyrate can be effluxed from colonocytes via MCT 1&4 and bind to GPR41, GPR43, and GPR109A (GPCRs) on other cells (such as dendritic cells) to exert immunomodulatory effects. Furthermore, intracellular butyrate can also act as histone deacetylase (HDAC) inhibitor, affecting proliferation and regulating the production of tight junction proteins and mucus. In summary butyrate can reduce inflammation, induce cell cycle inhibition, increase the expression of proteins that regulate gut barrier permeability, and increase mucus production. Made with BioRender.com.

Various SCFA transport systems have been identified, of which monocarboxylate transporter 1 (MCT1), MCT4, sodium-dependent monocarboxylate transporter (SMCT) 1 (SMCT1), and SMCT2 are most relevant for butyrate transport by colonocytes (Louis and Flint, 2017; Martin-Gallausiaux et al., 2021). MCT1 is expressed on both apical and basolateral membranes of colonocytes, and the uptake of butyrate takes place in combination with a proton (Figure 1) (Astbury and Corfe, 2012). The MCT4 transporter is only found on the basolateral membrane of colonocytes and therefore is responsible, in conjunction with MCT1, for efflux of butyrate into the blood (Sivaprakasam et al., 2017; Dalile et al., 2019). SMCT1 and SMCT2 in contrast are present only on the apical membrane of colonocytes (Astbury and Corfe, 2012; Dalile et al., 2019). Uptake by SMCT1 and SMCT2 is coupled to Na+ (with two and one Na + ions transported respectively). Butyrate uptake is dependent on the luminal concentration. Under normal conditions a majority is transported via MCT1 however, SMCT1 appears to dominate at low butyrate concentrations due to its high affinity for butyrate (Astbury and Corfe, 2012; Sivaprakasam et al., 2017).

After uptake by colonocytes, the majority of butyrate is used directly as an energy source and it represents up to 75% of the oxidative energy for these cells (Martin-Gallausiaux et al., 2021). Although much remains unknown about the intracellular transport of butyrate, the butyrate that has entered the cell eventually localizes in the mitochondria (Astbury and Corfe, 2012). Here, it is oxidized via β-oxidation to produce ATP (Astbury and Corfe, 2012). While this pathway is also possible for acetate and propionate, butyrate produces both more ATP upon oxidation and is oxidized at a faster rate than the other SCFAs due to butyrate not requiring the formation of intermediate species (Blaak et al., 2020). It is estimated that the energy obtained from SCFA oxidation accounts for 8% of the total daily energy needs of a human, demonstrating the vital role of microbiome metabolism (Astbury and Corfe, 2012; Blaak et al., 2020).

### 3.3 Butyrate and the gut–inflammation and barrier integrity

Beyond the use of butyrate directly as an energy source, butyrate can also affect the regulation of gut barrier integrity. TJ proteins form connections between the epithelial cells of the gut, ensuring the formation of a tight barrier between the cells (Morrison and Preston, 2016; Blaak et al., 2020; Karunaratne et al., 2020). *In vitro* studies using epithelial cells have shown that butyrate strengthens barrier integrity (Wang et al., 2012; Blaak et al., 2020). This was shown to be in principle due to an upregulation in the expression of the TJ protein claudin-1 and the redistribution of other TJ proteins (Zonnula occludens one and Occludin) in the cellular membrane (Wang et al., 2012). In fact, butyrate has been shown to improve epithelial barrier function after it has been disrupted due to lipopolysaccharide (LPS) exposure, demonstrating a possible restorative capacity of butyrate *in vitro* (Yan and Ajuwon, 2017). In addition to directly affecting the TJ proteins, butyrate can also increase the expression of the protein mucin 2 (MUC2) and hence further support a strong intestinal barrier by supporting the mucus layer in the GI tract (Liu et al., 2018).

Butyrate can also directly interact with a wide variety of cells via G-protein coupled receptors (GPCRs) (Liu et al., 2018; Martin-Gallausiaux et al., 2021; Salvi and Cowles, 2021). SCFAs are ligands for six GPCRs, of which GPR41 (FFAR3), GPR43 (FFAR2), and GPR109A (HCAR2) have a high affinity for butyrate (Liu et al., 2018; Martin-Gallausiaux et al., 2021). GPR41 and GPR43 are widely expressed in various organs (spleen, heart), tissues (muscle, colon epithelium), and immune cells (dendritic cells, regulatory T cells) (Liu et al., 2018; Dalile et al., 2019). Butyrate is the only SCFA to act as a ligand for GPR109A which is widely expressed in organs and immune cells (specifically dendritic cells and macrophages) (Liu et al., 2018; Martin-Gallausiaux et al., 2021). Butyrate reaches these receptors at other sites having been absorbed by colonocytes and travelling through the blood (Liu et al., 2018; Dalile et al., 2019).

Activation of these various GPCRs by butyrate leads to an anti-inflammatory effect via the upregulation of regulatory T cells and the production of anti-inflammatory cytokines (Interleukin (IL) 10 and Tumor growth factor β). This effect is mediated by both direct interaction with T cells via mammalian target of rapamycin (mTOR) activation and indirect effects by suppressing T cell activators (e.g., dendritic cells) (Dalile et al., 2019). Butyrate also acts via the suppression of pro-inflammatory cytokine production (Interferon γ, Tumor necrosis factor α, IL-1β, IL-6, and IL-8) through inhibition of NF-κB signaling in, for example, macrophages and endothelial cells (Aguilar et al., 2014; Liu et al., 2018). Another important effect of butyrate is its activity as an inhibitor of histone deacetylase (HDAC) (Figure 1) (Liu et al., 2018; Salvi and Cowles, 2021). With the alteration in gene transcription again leading to a reduction in inflammatory cytokine production by dendritic cells, T cells, and monocytes (Liu et al., 2018; Dalile et al., 2019). Altogether, these interactions mean that butyrate can create a tolerant environment within the mucosal immune system of the gut, but also systemically, by way of reducing inflammation and inflammatory factors (Figure 1) (Morrison and Preston, 2016; Dalile et al., 2019; Blaak et al., 2020). Altogether, butyrate is an important modulator of inflammation. Crucially for this review, inflammation is established to contribute to and accelerate αSyn aggregation and propagation thus having an important role in the development of PD (Kim et al., 2022).

## 4 Intestinal bacteria composition changes in PD

Composition of the intestinal bacteria in individuals with PD has been assessed in several studies (Hasegawa et al., 2015; Keshavarzian et al., 2015; Scheperjans et al., 2015; Unger et al., 2016; Bedarf et al., 2017; Hill-Burns et al., 2017; Hopfner et al., 2017; Li et al., 2017; 2019; Li et al., 2022 Z.; Mertsalmi et al., 2017; Minato et al., 2017; Petrov et al., 2017; Heintz-Buschart et al., 2018; Lin et al., 2018; 2019; Qian et al., 2018; Aho et al., 2019; Barichella et al., 2019; Pietrucci et al., 2019; Weis et al., 2019; Baldini et al., 2020; Cosma-Grigorov et al., 2020; Ren et al., 2020; Wallen et al., 2020; 2022; Zhang et al., 2020; Murros et al., 2021; Rosario et al., 2021; Takahashi et al., 2022; Nuzum et al., 2023) (see (Nuzum et al., 2020; Plassais et al., 2021; Romano et al., 2021; Papić et al., 2022) for review and metanalyses). Reporting of changes in the α-diversity of the PD microbiome is mixed. Several report no change in α-diversity measures (Scheperjans et al., 2015; Bedarf et al., 2017; Hopfner et al., 2017; Li et al., 2017; Lin et al., 2018; Aho et al., 2019; Pietrucci et al., 2019; Baldini et al., 2020; Nuzum et al., 2023), others report that α-diversity is increased in PD samples (an outgrowth of rare species and decrease in dominant species) (Keshavarzian et al., 2015; Qian et al., 2018; Barichella et al., 2019; Ren et al., 2020; Zhang et al., 2020; Li Z. et al., 2022), and some report the PD group to have a lower α-diversity compared to controls (Petrov et al., 2017; Cosma-Grigorov et al., 2020).

More consensus is reached regarding changes in β-diversity however, with studies consistently finding differences between PD and control groups regardless of differences in methodology, geographical location, and having accounted for potential confounding factors such as age and medication use (Nuzum et al., 2020; Romano et al., 2021; Papić et al., 2022). Specifically, a reduction of bacterial taxa typically associated with anti-inflammatory and neuroprotective effects was found, such as bacterial strains belonging to the *Lachnospiraceae* family (Barichella et al., 2019; Pietrucci et al., 2019; Vascellari et al., 2020; Romano et al., 2021; Papić et al., 2022). Additionally, bacteria that are typically associated as being beneficial (e.g., *Lactobacillus* and *Bifidobacterium*) were, perhaps counterintuitively, found to be more abundant in the PD microbiome (Hasegawa et al., 2015; Hill-Burns et al., 2017; Minato et al., 2017; Barichella et al., 2019; Nuzum et al., 2020; Romano et al., 2021; Papić et al., 2022; Takahashi et al., 2022) and in some studies were found to correlate with disease severity (Minato et al., 2017; Barichella et al., 2019; Baldini et al., 2020). This is partially attributed to these bacteria’s ability to survive in a proinflammatory environment (Romano et al., 2021) and their ability to outcompete other genera (Wallen et al., 2022). However it is also important to consider that this could also be due to methodological differences of some studies potentially biasing the outcome (e.g., failing to account for confounding use of probiotics, age, etc.) as explored in other reviews (Nuzum et al., 2020).

**FIGURE 2:**
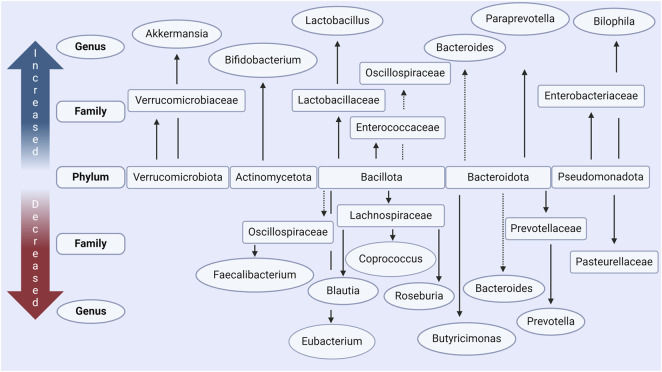
Main changes in gut microbiota in people with Parkinson’s disease according to *in vivo* data. Increased and decreased abundance is relative to case-controls in each respective study, in most studies these were age-matched. The main phyla which are commonly seen to be altered include *Verrucomicrobiota*, *Actinomycetota*, *Bacillota*, *Bacteroidota*, and *Pseudomondatoa*. Conflicting results, where some studies report increased levels and some decreased levels, are indicated with a dashed line. Made with BioRender.com.

At the phylum level, *Bacillota* is observed to correlate with disease duration in PD subjects in one study (Lin et al., 2018; Nuzum et al., 2020). In contrast, *Actinomycetota* and *Verrucomicrobiota* are present in a higher relative abundance (Figure 2) (Romano et al., 2021; Papić et al., 2022). However, findings for phylum level differences are often less conclusive due to some genera and some families of the same phylum observed to be more or less abundant in the PD microbiome within the same study. It has however often been found that bacteria of genus *Akkermansia* and *Bifidobacterium* (phylum *Verrucomicrobiota* and *Actinomycetota* respectively) are increased in prevalence in the PD microbiome (Keshavarzian et al., 2015; Unger et al., 2016; Bedarf et al., 2017; Hill-Burns et al., 2017; Lin et al., 2018; Nuzum et al., 2020; Romano et al., 2021) and correlate with the severity of motor (Heintz-Buschart et al., 2018; Papić et al., 2022) and non-motor (Li et al., 2019; Papić et al., 2022) symptoms. To find consistent differences in altered genera across studies is complex and conclusions vary due to differences in relative abundances being found, some of which directly contradict with others (Boertien et al., 2019; Papić et al., 2022; Wallen et al., 2022). This variability between findings and conclusions can be accredited to individual microbiome variations but can also largely be attributed to variation in experimental procedure (e.g., sequencing technique), small sample sizes, and/or lack of control of confounding variables (Nuzum et al., 2020; Papić et al., 2022; Wallen et al., 2022).

Critically in the context of this review, many of the bacteria decreased in PD are responsible for the production of SCFAs. It is therefore postulated that this reduces the total amount of SCFAs available in the GI tract (Bullich et al., 2019; Nuzum et al., 2020; 2023; Romano et al., 2021; Nishiwaki et al., 2022). This includes bacteria of the *Eubacterium* (Bedarf et al., 2017; Nuzum et al., 2020)*, Blautia* (Keshavarzian et al., 2015; Bedarf et al., 2017; Hill-Burns et al., 2017; Li et al., 2017; Lin et al., 2018; Nuzum et al., 2020; Romano et al., 2021; Papić et al., 2022; Takahashi et al., 2022)*, Roseburia* (Keshavarzian et al., 2015; Bedarf et al., 2017; Hill-Burns et al., 2017; Heintz-Buschart et al., 2018; Lin et al., 2018; Nuzum et al., 2020; Romano et al., 2021; Wallen et al., 2022)*, Faecalibacterium* (Unger et al., 2016; Hill-Burns et al., 2017; Li et al., 2017; Petrov et al., 2017; Lin et al., 2018; Nuzum et al., 2020)*,* and *Coprococcus* (Petrov et al., 2017; Nuzum et al., 2020) genera all of the phylum *Bacillota*. Bacteria of these genera are well established to be key producers of butyrate in the human microbiome (Nuzum et al., 2020). These findings are summarized in Figure 2 and Table 1. Similar reductions in SCFA producing bacteria are also seen in other neuro-inflammatory disorders (Cryan et al., 2020) as well as in Inflammatory Bowel disease (IBD) (Silva et al., 2018), where such changes are associated with an increased intestinal epithelial permeability and mucosal inflammation.

**TABLE 1:** Bacterial composition changes of relevance to butyrate production in clinical studies of PD and their subsequent measurement of butyrate levels. Changes reported are for PD cases compared to controls in fecal samples and are the reported changes seen after accounting for covariate factors (if performed) in each study. (NR; Not reported, OTUs; Operational taxonomic units, ACE; Abundance-based coverage estimators).

References	Α-diversity changes (index used)	Increased bacteria in PD samples	Decreased bacteria in PD samples	Butyrate levels in PD samples
Hasegawa et al. (2015)	NR	*Lactobacillus*	*Clostridium coccoides, C. leptum, Bacteroides fragilis*	NR
Keshavarzian et al. (2015)	Increase (Shannon, Gini-Simpson, Margalef)	*Akkermansia muciniphila*	*Blautia, Coprococcus, Roseburia*	NR
Scheperjans et al. (2015)	No change (Shannon, Inverse Simpson, Chao1, ACE)	-	*Prevotellaceae*	NR
Unger et al. (2016)	NR	*Bifidobacterium* sp.	*Bacteroidota, Faecalibacterium prausnitzii*	Reduced in stool
Bedarf et al. (2017)	No change (Richness, Shannon, Observed species, Chao1)	*Akkermansia muciniphila, Alistipes shahii*	*Eubacterium biforme, Lacrimispora saccharolytica, Segatella copri*	NR
Hill-Burns et al. (2017)	NR	*Akkermansia, Bifidobacterium*	*Blautia, Roseburia, Faecalibacterium*	NR (butyrate kinase predicted to be reduced)
Hopfner et al. (2017)	No change (Shannon, Simpson, Chao1, Faith’s phylogenetic distance)	*Barnesiellaceae, Enterococcaceae, Lactobacillaceae*	-	NR
Li et al. (2017)	No change (Shannon, Observed OTUs, Chao1)	*Actinomycetota*	*Blautia, Faecalibacterium, Ruminococcus*	NR
Petrov et al. (2017)	Decrease (Chao1)	*Lactobacillus, Bifidobacterium, Ruminococcus bromii*	*Faecalibacterium, Coprococcus, Bacteroides, Blautia*	NR
Heintz-Buschart et al. (2018)	NR	*Akkermansia*	*Roseburia*	NR (Predicted functionality showed no differences)
Lin et al. (2018)	No change (Shannon, Observed OTUs, Chao1, Phylogenetic diversity Whole tree)	*Akkermansia*	*Blautia, Roseburia, Faecalibacterium*	NR
Qian et al. (2018)	Increase (Shannon, Simpson, Observed species, Chao1, Phylogenetic diversity Whole tree)	*Lachnospiraceae, Butyricicoccus*	*Lactobacillus*	NR
Aho et al. (2019)	No change (Richness, Shannon, Inverse Simpson)	*-*	*Prevotella*	NR
Barichella et al. (2019)	Increase (Chao1)	-	Lachnospiraceae	NR
Li et al. (2019)	NR	*Akkermansia*	*Lactobacillus*	NR
Lin et al. (2019)	Increased (Shannon, Chao 1, Observed OTUs)	*Verrucomicrobia, Lactobacillus*	*Prevotella*	NR
Pietrucci et al. (2019)	No change (Shannon, Simpson, Chao1)	*Enterobacteriaceae, Enterococcaceae, Lactobacillaceae*	Lachnospiraceae	NR (Predicted increased metabolism of fatty acids)
Baldini et al. (2020)	No change (Shannon, Pielou evenness) Increase (Richness)	*Akkermansia muciniphila, Lactobacillus*	-	NR
Cosma-Grigorov et al. (2020)	Decrease (Simpson)	-	*Faecalibacterium*	NR
Ren et al. (2020)	Increase (Shannon, Simpson, Chao1, Phylogenetic diversity Whole tree, ACE)	*Blautia, Ruminococcus* (In normal cognition group)	*Blautia* (In mild cognitive impairment group)	No change
Wallen et al. (2020)	NR	*Bifidobacterium, Lactobacillus, Prevotella*	*Faecalibacterium, Blautia, Roseburia, Butyricicoccus*	NR
Vascellari et al. (2020)	No change (Richness)	*Akkermansia, Bifidobacterium, Clostridium*	*Blautia, Coprococcus, Lachnospira, Roseburia*	No change
Zhang et al. (2020)	Increase (Observed OTUs, Faith’s phylogenetic distance)	*Akkermansia*	*Fusobacterium*	NR
Li et al. (2022b)	Increase (Shannon, Simpson, Chao1, Observed species, Phylogenetic diversity Whole tree, ACE)	*Oscillospiraceae*	-	NR
Takahashi et al. (2022)	NR	*Lactobacillus*	*Blautia*	NR
Wallen et al. (2022)	NR	*Bifidobacterium dentium, Lactobacillus*	*Blautia, Roseburia, Eubacterium, Ruminococcus, Faecalibacterium prausnitzii*	NR (Predicted reduction in SCFA production)
Nuzum et al. (2023)	No change (Shannon, Simpson, Inverse Simpson, Observed species)	*Verrucomicrobiota, Bacillota*	-	NR (Predicted functionality showed no differences)

Intriguingly, some studies find increases in the family *Oscillospiraceae* in PD individuals*,* a family that is well established to contain several key butyrate producers (Qian et al., 2018; Ren et al., 2020; Li Z. et al., 2022). This could at first appear counterintuitive, however it is important to consider that a prominent butyrate producer of this family *Faecalibacterium prausnitzii* has been repeatedly shown to be reduced in the PD microbiota (Nuzum et al., 2020). Therefore it is plausible that other members of this family “overgrow” in the absence of *Faecalibacterium prausnitzii* without compensating for the loss of butyrate production and that this was not captured in these other studies. This further illustrates the need for next gen sequencing techniques that can provide detailed insight at a species level to elucidate this aspect of the PD microbiome.

The GI dysfunction seen in PD, often prodromal, could therefore plausibly be related to butyrate producing bacteria being lost and therefore GI microbiome homeostasis being disturbed which in turn can contribute to the early GI stages of PD and its progress. (Bullich et al., 2019; Nuzum et al., 2020). Furthermore, bacteria of the genus *Akkermansia,* seen to be increased in the PD microbiome (Keshavarzian et al., 2015; Bedarf et al., 2017; Hill-Burns et al., 2017; Heintz-Buschart et al., 2018; Lin et al., 2018; 2019; Li et al., 2019; Nuzum et al., 2020; Romano et al., 2021) are established to be mucin degraders which can therefore, in addition to the lack of butyrate stimulating mucin production, contribute to an increased intestinal epithelial permeability in PD (Nuzum et al., 2020; Nishiwaki et al., 2022).

A final important factor to consider is how such bacterial composition changes may evolve over time from early PD diagnosis toward later PD stages, particularly in regard to butyrate producing bacteria. On the whole, PD fecal samples are seen to increasingly diverge with respect to β-diversity from control samples with increasing disease duration (Barichella et al., 2019; Nuzum et al., 2020). More specifically, bacteria of the families *Lachnospiraceae* (Li et al., 2017; Barichella et al., 2019; Nuzum et al., 2020) and *Oscillospiraceae* (Li et al., 2017; Nuzum et al., 2020) were both negatively correlated with PD duration with an additional study showing decreases in the phylum *Bacillota* more generally (Keshavarzian et al., 2015; Nuzum et al., 2020). These families and the phylum *Bacillota* more broadly are well established to contain bacteria that contribute a significant amount of butyrate in the GI tract (Louis and Flint, 2017). Naturally, the classification of disease duration in such studies is precluded by the, in some cases, long prodromal phase of PD as previously discussed in this review as well as a variety of lifestyle factors that are known to influence gut bacteria composition. An alternative approach is a longitudinal design, which has been utilized in several studies (Minato et al., 2017; Aho et al., 2019; Hegelmaier et al., 2020; Lubomski et al., 2022). While similar changes in butyrate producers between healthy and PD subjects have been seen, this reduction does not seem to progress over time but rather remains low, even in patients where symptoms have deteriorated at follow up (Aho et al., 2019; Lubomski et al., 2022). Further studies of this kind, with longer follow up periods, are warranted to establish if this loss of butyrate produces progresses in a time-dependent manner.

## 5 Butyrate is often decreased in stool samples but not in the blood of subjects

Studies that have examined the microbiome of those with PD often also measure the SCFA content of the stool. The reduction in butyrate producing bacteria in the microbiome of those with PD is also sufficient to result in a lowered butyrate concentration in the stool of the same subjects when compared to age-matched controls (Unger et al., 2016; Tan et al., 2021; Chen et al., 2022; Voigt et al., 2022; Yang et al., 2022). This decreased level of butyrate in PD stool samples was also found to correlate to a variety of PD symptoms in several of these studies (Tan et al., 2021; Chen et al., 2022; Wu et al., 2022). Significant associations were found between decreased butyrate stool levels and worsening of postural instability-gait disorder symptoms (Tan et al., 2021); cognitive ability (Tan et al., 2021; Chen et al., 2022); motor performance (Chen et al., 2022); and depressive symptoms (Wu et al., 2022). Constipation was found to correlate with a decrease in fecal butyrate, but this did not remain significant after correcting for demographic and other features (Yang et al., 2022). Following correction, there was a correlation between reduced SCFAs other than butyrate in feces and an increase in blood SCFA concentrations (Yang et al., 2022).

Where studies on the fecal levels of butyrate find a consistent decrease across PD subjects, contradicting findings have been reported on blood levels of butyrate (Voigt et al., 2022; Yang et al., 2022). One study showed that blood levels were reduced; however, this was not related to the severity of the symptoms (Wu et al., 2022). Other studies discerned no significant difference in blood levels in butyrate of PD subjects compared to age-matched controls (Shin et al., 2020; Voigt et al., 2022; Yang et al., 2022). Intriguingly, one study determined an increase in blood levels of butyrate (Chen et al., 2022). It is hypothesized that increased serum SCFAs are a result of increased intestinal epithelial permeability something that is indeed perturbed in PD, although further research to confirm this is needed (Chen et al., 2022; Yang et al., 2022). This same reasoning can also possibly explain why fecal levels appear decreased but blood levels increased, again further research to determine this is also necessary (Chen et al., 2022). These findings suggest that fecal butyrate concentration possibly correlates more consistently to both motor and non-motor symptoms of PD than levels in the blood, but studies to specifically evaluate this are needed to establish if this is indeed a reliable finding.

### 5.1 Butyrate in preclinical models of PD

*In vivo* preclinical studies of PD have also led to interesting but limited number of observations regarding butyrate. It has been demonstrated that SCFAs, including butyrate, are actually elevated in fecal samples and contribute to disease progression in several studies of PD mice models (Sampson et al., 2016; Sun et al., 2018; Cannon et al., 2020). Particularly the studies of Sampson et al. and Cannon et al. present contradictory evidence for butyrate’s role in PD, with a majority of human studies showing, in fact, a decrease in fecal butyrate. It is interesting to note however, the ability of butyrate to firstly reach the brain via transporters on endothelial cells (Mitchell et al., 2011) and subsequently influence the activity of microglia cells within the brain showing that butyrate’s influence is not restricted to the GI tract (Sampson et al., 2016). However, this finding can be as a result of an as yet not understood therapeutic threshold for butyrate being exceeded which causes the normally beneficial SCFA to become, in fact, detrimental as demonstrated *in vitro* (Huang et al., 2014). One study that does replicate observations in humans is that of Turco et al. (Turco et al., 2023). It was found that the combination of antibiotic induced dysbiosis in the 6-hydroxydopamine model of PD resulted in not only a more severe onset of PD like symptoms but also a reduction in butyrate in the stool of the animals (Turco et al., 2023). It is therefore unclear how these findings implicate butyrate in preclinical models of PD and more investigation is warranted.

## 6 Therapeutic approaches exploiting butyrate in PD

### 6.1 Direct supplementation of sodium butyrate

Naturally, the correlation between butyrate and disease severity of PD has led to numerous investigations into butyrate supplementation, with sodium butyrate (NaB) the most commonly used form (Kratsman et al., 2016; Paiva et al., 2017; Srivastav et al., 2019; Qiao et al., 2020; Xu et al., 2022; Zhang et al., 2022). *In vitro* studies found NaB to exert neuroprotective effects protecting against αSyn and other toxins (Paiva et al., 2017; Getachew et al., 2020; Zhang et al., 2022). In general, the protective mechanism is attributed to NaB both regulating gene expression due to its HDAC activity and regulation of gene repair (Paiva et al., 2017; Getachew et al., 2020). Interestingly, this protective effect of NaB was not seen with other SCFAs in rotenone-induced toxicity *in vitro* studies (Zhang et al., 2022). This again was attributed to the ability of NaB to alter the autophagic response to toxic αSyn species via HDAC inhibition and therefore protect dopaminergic neurons (Zhang et al., 2022). When investigating the neurotoxicity of αSyn in DA cells it was discovered that DNA damage caused by αSyn was associated with reduced acetylation of histone H3 (Paiva et al., 2017). Treatment with NaB resulted in the rescue of αSyn-induced damage, due to its ability to alter HDAC activity (Paiva et al., 2017). Furthermore, the ability of NaB to act as an agonist to GPR41 led to less dopaminergic cell death *in vitro*, further demonstrating that NaB acts in a multifaceted manner (Getachew et al., 2020).

In animal models of PD, the administration of NaB leads to improvement of various PD-like symptoms, although there is discussion on the accountable mechanism of action (St Laurent et al., 2013; Sharma et al., 2015; Liu et al., 2017; Srivastav et al., 2019; Qiao et al., 2020; Kakoty et al., 2021; Xu et al., 2022; Guo et al., 2023; Zhang et al., 2023) (Studies are summarized in Table 2). In various mouse and rat models of PD, NaB treatment results in amelioration of systemic inflammation, improvements in motor deficits, improvement of intestinal barrier function, reduction in microglia activation, and an elevation of dopamine levels (Sharma et al., 2015; Liu et al., 2017; Srivastav et al., 2019; Kakoty et al., 2021; Xu et al., 2022; Guo et al., 2023; Zhang et al., 2023). In a 1-methyl-4-phenyl-1,2,3,6-tetrahydropyridine (MPTP) based mouse model of PD higher levels of epithelial TJ proteins were found after NaB treatment, consequently rescuing the intestinal epithelial barrier that was otherwise disrupted (Xu et al., 2022). As the effects of NaB treatment were similar to treatment with a GPRR109A ligand (monomethyl fumarate), the therapeutic influence of butyrate was accredited to its potential as a GPR109A agonist (Xu et al., 2022). On the contrary, two studies investigating NaB treatment in different rat models (Pre-formed α-Syn fibrils and 6-hydroxydopamine) attributed the observed therapeutic effects to the HDAC inhibitory potential of NaB, as both studies observed symptom improvement with a corresponding increase in histone H3 acetylation (Sharma et al., 2015; Kakoty et al., 2021). It is perfectly plausible that more than one pathway is responsible for the neuroprotective and anti-inflammatory effects of NaB treatment. In a study that investigated the immunoregulatory effect of butyrate in an *in vitro* model in the context of IBD based on its potential as a GPR43 ligand, it was demonstrated that its protection could not be matched with the administration of a single other GPR43 agonist (D’Souza et al., 2017). Similarly, the mediative effects of NaB in cellular and animal models of PD might be caused by both its potential as GPR41, GPR43 and GPR109A ligand as well as its activity as an HDAC inhibitor.

**TABLE 2:** Findings of interventional studies using sodium butyrate, prebiotics and probiotics in preclinical and clinical studies. NaB; Sodium butyrate, i.p; Intraperitoneal, i.g.; intragastric, MPTP; 1-methyl-4-phenyl-1,2,3,6-tetrahydropyridine, PFF; pre-formed fibrils, NR; Not reported, DHA; docosahexaenoic acid, SCFA; Short-chain fatty acid.

References	Study population (PD model)	Intervention	Motor symptoms	Non-motor symptoms	Other
St Laurent et al. (2013)	*Drosophila* (rotenone exposure)	NaB (10 mM in food)	Reduced motor impairment	-	-
Sharma et al. (2015)	Rat (6-OHDA intrastriatal lesion)	NaB (up to 300 mg/kg/day i.p. for 14 days)	Reduced motor impairment	-	Increased striatal dopamine level, reduced neuroinflammatory markers
Liu et al. (2017)	Mouse (MPTP i.p. injection)	NaB (200 mg/kg/day i.g. for 3 weeks)	Reduced motor impairment	Reduced depressive symptoms	Reduced loss of dopaminergic neurons
Srivastav et al. (2019)	Mouse (MPTP i.p. injection)	NaB (200 mg/kg i.g. 3x per week for 2 weeks)	-	-	Reduced loss of dopaminergic neurons
Qiao et al. (2020)	Mouse (MPTP i.p. injection)	NaB (165 mg/kg/day i.g. for 3 weeks)	Worsening of motor deficits	Increased inflammation in colon	Increased loss of dopaminergic neurons
Kakoty et al. (2021)	Rat (PFF α-Syn injection)	NaB (300 mg/kg i.g.)	Reduced motor impairment	Reduced intestinal inflammation	-
Xu et al. (2022)	Mouse (MPTP i.p. injection)	NaB (600 mg/kg/day i.g. for 2 weeks	Reduced motor impairment	Reduced intestinal inflammation	Reduced loss of dopaminergic neurons
Guo et al. (2023)	Mouse (MPTP i.p. injection)	NaB (NR)	Reduced motor impairment	Reduced intestinal inflammation	Reduced loss of dopaminergic neurons
Zhang et al. (2023)	Mouse (Rotenone i.g.)	NaB (200 mg/kg/day for 4 weeks)	Reduced motor impairment	Reduced intestinal inflammation	Reduced loss of dopaminergic neurons and α-Syn aggregation
Perez-Pardo et al. (2017)	Mouse (Rotenone intrastriatal lesion)	Dietary intervention containing uridine and DHA (4 weeks post model induction)	Reduced motor impairment	Reduced intestinal inflammation, Reduced cognitive impairment	No change in number of dopaminergic neurons
Perez-Pardo et al. (2018)	Mouse (Rotenone intrastriatal lesion)	Dietary intervention containing uridine, DHA, and prebiotic fibers (4 weeks post model induction)	Reduced motor impairment	Improved spatial memory, improved intestinal transit	-
Srivastav et al. (2019)	Mouse (MPTP i.p. injection) and Mouse (Rotenone i.p.)	*Lactobacillus rhamnosus* GG (L-GG)*, Bifidobacterium animalis lactis* (BB-12), and *Lactobacillus acidophilus* (LA-5) in drinking water for 30 days	Reduced motor impairment	-	Reduced loss of dopaminergic neurons
Li et al. (2022a)	Mouse (MPTP i.p. injection)	*Bifidobacterium breve* CCFM1067 (10^9^ CFU/200 μL i.g. daily for 30 days) *NB* prior to model induction	Reduced motor impairment	-	Increase in intestinal butyrate
Pan et al. (2022)	Mouse (MPTP i.p. injection)	*Pediococcus pentosaceus* WMU002 (2 × 10^8^ CFU/day i.g. for 4 weeks)	Reduced motor impairment	-	Reduced loss of dopaminergic neurons and α-Syn aggregation
Barichella et al. (2016)	PD individuals with constipation	Fermented milk, containing multiple probiotic strains and prebiotic fiber	-	Increased number of complete bowel movements	-
Hegelmaier et al. (2020)	PD (non-monogenic form)	Bowel cleansing + vegetarian diet	UPDRS III decrease	UPDRS III decrease	Decreased levodopa-equivalent daily dose
Becker et al. (2022)	PD (Any diagnosis)	RS prebiotic fiber (5g twice daily, oral)	-	No changes in bowel habits, reduced depressive symptoms	Increase in fecal butyrate
Du et al. (2022)	PD individuals with constipation	*Bacillus licheniformis* (2.5 × 10^9^ CFU, 2 capsules 3x per day) + *Lactobacillus acidophilus, Bifidobacterium longum, Enterococcus faecalis* (BIFICO, 1.0 × 10^7^ CFU/strain, 4 capsules, 2x per day) for 12 weeks	-	Increased number of complete bowel movements	-
Hall et al. (2023)	PD individuals (medicated and non-medicated)	Prebiotic bar (containing resistant starch, rice brain, resistant maltodextrin, and inulin) for 10 days	Decreased UPDRS score from baseline	Decreased UPDRS score from baseline, reduced plasma zonulin	Increase in SCFA producing bacteria

Importantly, not all studies that investigated the administration of NaB in PD models found a beneficial effect. In another use of the MPTP mouse model of PD, NaB supplementation resulted in a significant aggravation of motor deficits, an increase in neuroinflammation and colon inflammation, and a decrease in dopamine levels and DA neurons (Qiao et al., 2020). As the oral dose of NaB in this study is comparable to the ones that described a protective role of butyrate and it uses a similar model to the other studies, this contradiction is yet to be explained. As one *in vitro* study found that butyrate restored intestinal barrier function in low doses but increased barrier dysfunction in higher doses due to apoptotic effects, it has been demonstrated that the beneficial effect of butyrate is concentration-dependent (Huang et al., 2014). In support of this, butyrate supplementation in a mouse model for autism led to improved social deficits in lower doses but did not have significant effects in high doses (Kratsman et al., 2016).

Not all studies that examine butyrate observe a reduction in its serum concentration (Voigt et al., 2022; Yang et al., 2022). Therefore, oral administration of NaB is most likely to exert its effect via the gut and the colonocytes and perhaps not via systemic mechanisms. Additionally, the dosing of NaB is an item of debate, as the daily demand of butyrate in physiological conditions has a wide suspected range (Banasiewicz et al., 2020). Individual demand may, therefore, differ depending on lifestyle, diet, and age, which complicates translation from animal models to humans (Banasiewicz et al., 2020).

### 6.2 Pro/pre biotics to supplement butyrate

In addition to directly supplementing the depleted butyrate by NaB, it is also possible to “steer” the altered microbiota composition to produce more butyrate itself via the use of pro- and prebiotics. This can have a complimentary effect in that the butyrate levels are restored in addition to suppressing the outgrowth of other non-beneficial and sometimes inflammatory bacteria. After the administration of probiotics, prebiotic fibers (including oligosaccharides), or a high-fiber diet several studies investigated the gut microbial changes and the consequent effects on PD symptoms (Perez-Pardo et al., 2017; 2018; Srivastav et al., 2019; Ghyselinck et al., 2021; Li T. et al., 2022; Pan et al., 2022) (Studies are summarized in Table 2).

In animal models of PD, the supplementation of various probiotics, all belonging to the phylum Bacillota, resulted in gut microbiota composition alterations and is associated with increased levels of fecal butyrate (Srivastav et al., 2019; Li T. et al., 2022; Pan et al., 2022). When investigating the neuroprotective effects of this supplementation in the brain, it was found that DA neurons were protected from cell death, glial cell hyperactivation was suppressed, and neuroinflammation was reduced (Srivastav et al., 2019; Li T. et al., 2022). In addition, the blood-brain barrier and intestinal epithelial barrier were protected from damage associated with an MPTP-induced PD mouse model (Li T. et al., 2022) and in an *in vitro* model (Ghyselinck et al., 2021). These therapeutic effects were generally accredited to bacterial butyrate levels, as an increase in fecal and brain levels of butyrate was correlated to neuroprotective effects and administration of NaB resulted in a similar disease amelioration (Srivastav et al., 2019; Li T. et al., 2022). A similar effect was seen when a prebiotic diet was used in two other studies, whereby PD like symptoms including motor dysfunction and spatial memory deficit were improved by addition of prebiotic oligosaccharides (Perez-Pardo et al., 2017; 2018). Though it was not directly measured, in both cases the additional benefit of the prebiotic fibers was attributed to the microbiota modulatory effects in addition to their direct effects on immune and GI functions (Perez-Pardo et al., 2017; 2018).

One study, however, attributed the motor symptom improvement to the bacterial metabolite γ-aminobutyric acid (GABA), as increased GABA levels were observed in the brains of treated mice compared to the PD model (Pan et al., 2022). This metabolite is predominantly produced by bacteria belonging to the *Bacteroides* genus, of which the relative abundance is found to be increased in some studies or decreased in others with regard to PD microbiome (Nuzum et al., 2020; Romano et al., 2021). It however remains controversial if GABA produced in the intestines by bacteria can pass the blood brain barrier (Boonstra et al., 2015; Mazzoli and Pessione, 2016).

Besides animal studies, investigations of high-fiber/prebiotic fiber diets and/or probiotic supplementation in humans have been performed (Barichella et al., 2016; Hegelmaier et al., 2020; Baert et al., 2021; Becker et al., 2022; Du et al., 2022; Hall et al., 2023) (Summarized in Table 2). Baert *et al.* (2021) examined the effect of 40 separate fiber supplementations (including inulins, oligosaccharides, and resistant starch) on the human gut microbiota *in vitro* and associated bacterial butyrate levels in the stool of PD subjects and age-matched controls. Although all fiber supplementations led to an increase in butyrate production *in vitro*, PD subjects’ microbiota consistently produced less butyrate than age-matched controls. Another study that examined the effect of a resistant starch diet in PD found an increase in fecal butyrate levels of PD subjects, compared to PD subjects that received solely dietary instructions (Becker et al., 2022). This was associated with a significant improvement in non-motor symptoms (measured on the non-motor symptoms questionnaire (NMSQ)) and depressive symptoms but not with constipation (Becker et al., 2022). This lack of improvement in constipation was attributed to the relatively short intervention period of this study (8 weeks) compared to other studies regarding resistant starches in mid-age and elderly subjects (12 weeks) (Alfa et al., 2018; Becker et al., 2022).

Probiotics have also been seen to have a benefit regarding non-motor symptoms of PD, particularly constipation and bloating (Cassani et al., 2011; Georgescu et al., 2016) but also on PD symptoms more generally based on the Movement Disorders Society-Unified Parkinson’s Disease Rating Scale (MDS-UPDRS) (Tamtaji et al., 2019). Probiotics used in these studies were varied but included: *Lacticaseibacillus* casei Shirota (Cassani et al., 2011); *Lactobacillus* acidophilus and *Bifidobacterium infantis* (Georgescu et al., 2016); *Lactobacillus* acidophilus, *Bifidobacterium bifidum*, *Limosilactobacillus reuteri*, and *Limosilactobacillus fermentum* (Tamtaji et al., 2019). These studies give a promising first look into the effectiveness of probiotics and trials using larger populations should be conducted to examine if similar results can be found when more participants are included.

An investigation of the impact of a vegetarian diet in combination with bowel cleansing in people with PD and healthy controls compared symptoms and levodopa therapy response after treatment (Hegelmaier et al., 2020). It was found that the gut microbiota composition notably changed after both treatments, which was associated with a significant improvement in motor symptoms. In a 1-year follow-up, the levodopa-equivalent daily dose was decreased in both treatment groups indicating that dietary intervention or bowel cleansing may offer supplementary, non-pharmacological treatments for PD subjects (Hegelmaier et al., 2020). However, while microbiota composition was evaluated and trends toward changes in butyrate producers were seen, there was no direct measurement of butyrate in this study which could have an added value in explaining the mechanism for this effect (Hegelmaier et al., 2020). Finally, in an open label study, supplementation with prebiotic fibers (resistant starch, rice brain, resistant maltodextrin, and inulin) was sufficient to induce positive changes in gut microbiota composition (an increase of SCFA producers), as well as exploratory analyses showing promising trends in other clinical outcomes such as GI discomfort and intestinal permeability (Hall et al., 2023).

### 6.3 Fecal microbiota transplantation

Fecal microbiota transplantation (FMT) takes the microbiota of a healthy donor and transfers this into the recipient but its use in PD is thus far limited (Huang et al., 2019; Segal et al., 2021; DuPont et al., 2023). Preclinical studies have shown positive results with protective effects in two separate studies (Sun et al., 2018; Zhao et al., 2021). The improvement in PD-like symptoms in the MPTP and rotenone induced mouse models of PD were attributed to a reduction in inflammatory pathways however not directly to increased fecal butyrate (Sun et al., 2018; Zhao et al., 2021) In clinical studies it has been found that FMT is most effective in improving non-motor symptoms (constipation, sleep impairment) with limited results reporting improvements in motor symptoms (Segal et al., 2021; DuPont et al., 2023). Interestingly, butyrate producing bacteria were seen to reestablish themselves in the microbiome of PD patients after receiving transplant suggesting that FMT could present a viable method to restore the microbiota production of SCFAs (DuPont et al., 2023). In general, FMT appears to be well tolerated in PD patients with minimal adverse reaction (DuPont et al., 2023). Larger trials are being conducted but as of writing, no results other than that FMT is safe and tolerated in PD are available (Vendrik et al., 2023).

## 7 Discussion

The exact involvement of butyrate in PD is still to be unraveled. On the one hand, many studies have reported changes in the microbiome of those with PD characterized by a loss of butyrate producing bacteria (Bullich et al., 2019; Nuzum et al., 2020; Romano et al., 2021). The potential of butyrate to regulate important epithelial TJ proteins and inflammatory processes in the gut and the brain is a key reason why the loss of butyrate producing bacteria may contribute to PD development as well as symptom severity (Dutta et al., 2019; Tan et al., 2022). Particularly of note is the ability of butyrate to regulate inflammation as this is especially relevant in the context of the aggregation and propagation of αSyn, a key hallmark of PD (Kim et al., 2022). Further evidence is found in preclinical studies where probiotic supplementation resulted in elevated butyrate levels and protected against neurodegeneration (Srivastav et al., 2019) as well as *in vitro* studies where butyrate could protect against neurotoxicity (Zhang et al., 2022).

On the other hand, findings are not always consistent in addition to little evidence being found for butyrate being depleted in the blood of those with PD and hence it is not well understood if the amount of butyrate reaching the brain would also be altered. It is also important to consider that GI levels of butyrate may not be relevant in all cases of PD with some possible subtypes hypothesized to have little GI involvement (Borghammer and Van Den Berge, 2019). Furthermore, it has also been seen in preclinical studies that butyrate can in fact have a detrimental effect on PD-like symptoms (Sampson et al., 2016; Qiao et al., 2020). This discrepancy is difficult to explain but can, in part, be as a result of heterogeneous preclinical models for PD being used as well as differences in the amount of butyrate administered compared to studies where positive effects were observed.

Crucially, it is unknown whether butyrate has a causative role in PD or is merely a result of a more generalized gut bacteria disruption. Many of the studies performed show strong correlative relationships between fecal butyrate levels and symptom severity, but so far a causal relationship has proved difficult to find.

This review also aimed to examine the potential of butyrate as a therapeutic in PD. Several *in vivo* studies have assessed NaB as a supplement to alter GI butyrate levels (Qiao et al., 2020; Kakoty et al., 2021; Xu et al., 2022; Guo et al., 2023). Although promising, these have not yet progressed beyond preclinical studies. The use of NaB to alter butyrate levels in the GI tract therefore remains underexplored in the context of PD and more investigation is warranted into this safe and well tolerated potential treatment (Banasiewicz et al., 2020). Pre- and probiotics have been more thoroughly evaluated with short duration trials finding improvements in GI related symptoms and some motor-related outcomes in humans (Du et al., 2022; Hall et al., 2023). Whilst these improvements are not necessarily as a direct result of butyrate levels being restored it is an important factor that should be evaluated in future studies. It also remains to be seen if, on longer time scales, these benefits in motor symptoms and GI related symptoms remain. Finally, the use of FMT in PD to restore changes in the microbiota and consequent butyrate levels is in its infancy in PD but it has gained traction in other fields (Van Laar et al., 2019). Preliminary studies in PD seem to be well tolerated as well as able to effectively bolster the reduced populations of butyrate producers (DuPont et al., 2023). Whether this is a more effective approach than supplementation of butyrate or pre- and probiotics is not known, and therefore the potential applications of FMT in PD need to be investigated more thoroughly.

Another important future consideration is the safety and regulation of pre-/probiotics. While studies show that they can be well tolerated, there is the possibility for unintended interactions to arise, particularly in the context of PD. As previously mentioned, some strains of bacteria possess enzymes that can alter the efficacy of PD medication (namely, Levodopa) (van Kessel et al., 2019; Zhong et al., 2023). This is true for some strains that are included in over-the-counter probiotic supplements and therefore caution should be exercised in their use (van Kessel et al., 2019). Furthermore, one butyrate producer, *Clostridium sporogenes,* is also capable of modifying the structure of Levodopa (Guo et al., 2019) and therefore future studies should be vigilant to ensure medication effectiveness is not altered by a change in the microbiome (Zhong et al., 2023).

In conclusion, the role of butyrate in PD is complex. Further difficulty comes in understanding whether butyrate depletion is a cause or an effect of PD. Clarity will be gained by future studies if butyrate levels are assessed both in the feces and in the blood of participants as this will aid in giving a more complete picture of how this may correlate with disease severity and outcomes. Furthermore, more investigation as to whether butyrate is involved in the very earliest stages of the disease are also necessary to discern if butyrate plays a causative role or is merely associated with disease severity post disease onset. This however remains difficult due to the sometimes long prodromal phase associated with PD.

## References

[B1] Aguilar E. C. Leonel A. J. Teixeira L. G. Silva A. R. Silva J. F. Pelaez J. M. N. (2014). Butyrate impairs atherogenesis by reducing plaque inflammation and vulnerability and decreasing NFκB activation. Nutr. Metabolism Cardiovasc. Dis. 24, 606–613. 10.1016/j.numecd.2014.01.002 24602606

[B2] Aho V. T. E. Houser M. C. Pereira P. A. B. Chang J. Rudi K. Paulin L. (2021). Relationships of gut microbiota, short-chain fatty acids, inflammation, and the gut barrier in Parkinson’s disease. Mol. Neurodegener. 16, 6. 10.1186/s13024-021-00427-6 33557896 PMC7869249

[B3] Aho V. T. E. Pereira P. A. B. Voutilainen S. Paulin L. Pekkonen E. Auvinen P. (2019). Gut microbiota in Parkinson’s disease: temporal stability and relations to disease progression. EBioMedicine 44, 691–707. 10.1016/j.ebiom.2019.05.064 31221587 PMC6606744

[B4] Alfa M. J. Strang D. Tappia P. S. Graham M. Domselaar G. V. Forbes J. D. (2018). A randomized trial to determine the impact of a digestion resistant starch composition on the gut microbiome in older and mid-age adults. Clin. Nutr. 37, 797–807. 10.1016/j.clnu.2017.03.025 28410921

[B5] Astbury S. M. Corfe B. M. (2012). Uptake and metabolism of the short-chain fatty acid butyrate, a critical review of the literature. Curr. Drug Metab. 13, 815–821. 10.2174/138920012800840428 22571479

[B6] Baert F. Matthys C. Maselyne J. Van Poucke C. Van Coillie E. Bergmans B. (2021). Parkinson’s disease patients’ short chain fatty acids production capacity after *in vitro* fecal fiber fermentation. NPJ Park. Dis. 7, 72. 10.1038/s41531-021-00215-5 PMC8363715 34389734

[B7] Baldini F. Hertel J. Sandt E. Thinnes C. C. Neuberger-Castillo L. Pavelka L. (2020). Parkinson’s disease-associated alterations of the gut microbiome predict disease-relevant changes in metabolic functions. BMC Biol. 18, 62. 10.1186/s12915-020-00775-7 32517799 PMC7285525

[B8] Banasiewicz T. Domagalska D. Borycka-Kiciak K. Rydzewska G. (2020). Determination of butyric acid dosage based on clinical and experimental studies - a literature review. Prz. Gastroenterol. 15, 119–125. 10.5114/pg.2020.95556 32550943 PMC7294979

[B9] Barichella M. Pacchetti C. Bolliri C. Cassani E. Iorio L. Pusani C. (2016). Probiotics and prebiotic fiber for constipation associated with Parkinson disease: an RCT. Neurology 87, 1274–1280. 10.1212/WNL.0000000000003127 27543643

[B10] Barichella M. Severgnini M. Cilia R. Cassani E. Bolliri C. Caronni S. (2019). Unraveling gut microbiota in Parkinson’s disease and atypical parkinsonism. Mov. Disord. 34, 396–405. 10.1002/mds.27581 30576008

[B11] Becker A. Schmartz G. P. Gröger L. Grammes N. Galata V. Philippeit H. (2022). Effects of resistant starch on symptoms, fecal markers, and gut microbiota in Parkinson’s disease — the RESISTA-PD trial. Genomics, Proteomics Bioinforma. 20, 274–287. 10.1016/j.gpb.2021.08.009 PMC9684155 34839011

[B12] Bedarf J. R. Hildebrand F. Coelho L. P. Sunagawa S. Bahram M. Goeser F. (2017). Functional implications of microbial and viral gut metagenome changes in early stage L-DOPA-naïve Parkinson’s disease patients. Genome Med. 9, 39. 10.1186/s13073-017-0428-y 28449715 PMC5408370

[B13] Blaak E. E. Canfora E. E. Theis S. Frost G. Groen A. K. Mithieux G. (2020). Short chain fatty acids in human gut and metabolic health. Benef. Microbes 11, 411–455. 10.3920/BM2020.0057 32865024

[B14] Bloch A. Probst A. Bissig H. Adams H. Tolnay M. (2006). Alpha-synuclein pathology of the spinal and peripheral autonomic nervous system in neurologically unimpaired elderly subjects. Neuropathology Appl. Neurobiol. 32, 284–295. 10.1111/j.1365-2990.2006.00727.x 16640647

[B15] Boertien J. M. Murtomäki K. Pereira P. A. B. van der Zee S. Mertsalmi T. H. Levo R. (2022). Fecal microbiome alterations in treatment-naive *de novo* Parkinson’s disease. NPJ Park. Dis. 8, 129. 10.1038/s41531-022-00395-8 PMC9551094 36216843

[B16] Boertien J. M. Pereira P. A. B. Aho V. T. E. Scheperjans F. (2019). Increasing comparability and utility of gut microbiome studies in Parkinson’s disease: a systematic review. J. Park. Dis. 9, S297–S312. 10.3233/JPD-191711 PMC6839453 31498131

[B17] Boonstra E. de Kleijn R. Colzato L. S. Alkemade A. Forstmann B. U. Nieuwenhuis S. (2015). Neurotransmitters as food supplements: the effects of GABA on brain and behavior. Front. Psychol. 6, 1520. 10.3389/fpsyg.2015.01520 26500584 PMC4594160

[B18] Borghammer P. Van Den Berge N. (2019). Brain-first versus gut-first Parkinson’s disease: a hypothesis. J. Park. Dis. 9, S281–S295. 10.3233/JPD-191721 PMC6839496 31498132

[B19] Braak H. de Vos R. A. I. Bohl J. Del Tredici K. (2006). Gastric alpha-synuclein immunoreactive inclusions in Meissner’s and Auerbach’s plexuses in cases staged for Parkinson’s disease-related brain pathology. Neurosci. Lett. 396, 67–72. 10.1016/j.neulet.2005.11.012 16330147

[B20] Braak H. Tredici K. D. Rüb U. de Vos R. A. I. Jansen Steur E. N. H. Braak E. (2003). Staging of brain pathology related to sporadic Parkinson’s disease. Neurobiol. Aging 24, 197–211. 10.1016/S0197-4580(02)00065-9 12498954

[B21] Bullich C. Keshavarzian A. Garssen J. Kraneveld A. Perez-Pardo P. (2019). Gut vibes in Parkinson’s disease: the microbiota-gut-brain Axis. Mov. Disord. Clin. Pract. 6, 639–651. 10.1002/mdc3.12840 31745471 PMC6856467

[B22] Cannon T. Sinha A. Trudeau L.-E. Maurice C. F. Gruenheid S. (2020). Characterization of the intestinal microbiota during Citrobacter rodentium infection in a mouse model of infection-triggered Parkinson’s disease. Gut Microbes 12, 1–11. 10.1080/19490976.2020.1830694 PMC7575009 33064969

[B23] Cassani E. Privitera G. Pezzoli G. Pusani C. Madio C. Iorio L. (2011). Use of probiotics for the treatment of constipation in Parkinson’s disease patients. Minerva Gastroenterol. Dietol. 57, 117–121. 21587143

[B24] Chen S.-J. Chen C.-C. Liao H.-Y. Lin Y.-T. Wu Y.-W. Liou J.-M. (2022). Association of fecal and plasma levels of short-chain fatty acids with gut microbiota and clinical severity in patients with Parkinson disease. Neurology 98, e848–e858. 10.1212/WNL.0000000000013225 34996879 PMC8883514

[B25] Chiang H.-L. Lin C.-H. (2019). Altered gut microbiome and intestinal pathology in Parkinson’s disease. J. Mov. Disord. 12, 67–83. 10.14802/jmd.18067 31158941 PMC6547039

[B26] Clairembault T. Leclair-Visonneau L. Coron E. Bourreille A. Le Dily S. Vavasseur F. (2015). Structural alterations of the intestinal epithelial barrier in Parkinson’s disease. Acta Neuropathol. Commun. 3, 12. 10.1186/s40478-015-0196-0 25775153 PMC4353469

[B27] Cosma-Grigorov A. Meixner H. Mrochen A. Wirtz S. Winkler J. Marxreiter F. (2020). Changes in gastrointestinal microbiome composition in PD: a pivotal role of covariates. Front. Neurol. 11, 1041. 10.3389/fneur.2020.01041 33071933 PMC7538808

[B28] Cresci G. A. Bawden E. (2015). Gut microbiome: what we do and don’t know. Nutr. Clin. Pract. 30, 734–746. 10.1177/0884533615609899 26449893 PMC4838018

[B29] Cryan J. F. O’Riordan K. J. Sandhu K. Peterson V. Dinan T. G. (2020). The gut microbiome in neurological disorders. Lancet Neurol. 19, 179–194. 10.1016/S1474-4422(19)30356-4 31753762

[B30] Cui H. Elford J. D. Alitalo O. Perez-Pardo P. Tampio J. Huttunen K. M. (2023). Nigrostriatal 6-hydroxydopamine lesions increase alpha-synuclein levels and permeability in rat colon. Neurobiol. Aging 129, 62–71. 10.1016/j.neurobiolaging.2023.05.007 37271045

[B31] Culp E. J. Goodman A. L. (2023). Cross-feeding in the gut microbiome: ecology and mechanisms. Cell Host Microbe 31, 485–499. 10.1016/j.chom.2023.03.016 37054671 PMC10125260

[B32] Dalile B. Van Oudenhove L. Vervliet B. Verbeke K. (2019). The role of short-chain fatty acids in microbiota–gut–brain communication. Nat. Rev. Gastroenterol. Hepatol. 16, 461–478. 10.1038/s41575-019-0157-3 31123355

[B33] den Besten G. van Eunen K. Groen A. K. Venema K. Reijngoud D.-J. Bakker B. M. (2013). The role of short-chain fatty acids in the interplay between diet, gut microbiota, and host energy metabolism. J. Lipid Res. 54, 2325–2340. 10.1194/jlr.R036012 23821742 PMC3735932

[B34] Dexter D. T. Jenner P. (2013). Parkinson disease: from pathology to molecular disease mechanisms. Free Radic. Biol. Med. 62, 132–144. 10.1016/j.freeradbiomed.2013.01.018 23380027

[B35] D’Souza W. N. Douangpanya J. Mu S. Jaeckel P. Zhang M. Maxwell J. R. (2017). Differing roles for short chain fatty acids and GPR43 agonism in the regulation of intestinal barrier function and immune responses. PLoS One 12, e0180190. 10.1371/journal.pone.0180190 28727837 PMC5519041

[B36] Du Y. Li Y. Xu X. Li R. Zhang M. Cui Y. (2022). Probiotics for constipation and gut microbiota in Parkinson’s disease. Park. Relat. Disord. 103, 92–97. 10.1016/j.parkreldis.2022.08.022 36087572

[B37] DuPont H. L. Suescun J. Jiang Z.-D. Brown E. L. Essigmann H. T. Alexander A. S. (2023). Fecal microbiota transplantation in Parkinson’s disease—a randomized repeat-dose, placebo-controlled clinical pilot study. Front. Neurology 14, 1104759. 10.3389/fneur.2023.1104759 PMC10019775 36937520

[B38] Dutta S. K. Verma S. Jain V. Surapaneni B. K. Vinayek R. Phillips L. (2019). Parkinson’s disease: the emerging role of gut dysbiosis, antibiotics, probiotics, and fecal microbiota transplantation. J. Neurogastroenterol. Motil. 25, 363–376. 10.5056/jnm19044 31327219 PMC6657920

[B39] Georgescu D. Ancusa O. E. Georgescu L. A. Ionita I. Reisz D. (2016). Nonmotor gastrointestinal disorders in older patients with Parkinson’s disease: is there hope? Clin. Interventions Aging 11, 1601–1608. 10.2147/CIA.S106284 PMC5113937 27956826

[B40] Getachew B. Csoka A. B. Bhatti A. Copeland R. L. Tizabi Y. (2020). Butyrate protects against salsolinol-induced toxicity in SH-SY5Y cells: implication for Parkinson’s disease. Neurotox. Res. 38, 596–602. 10.1007/s12640-020-00238-5 32572814 PMC7484007

[B41] Ghaisas S. Maher J. Kanthasamy A. (2016). Gut microbiome in health and disease: linking the microbiome-gut-brain axis and environmental factors in the pathogenesis of systemic and neurodegenerative diseases. Pharmacol. Ther. 158, 52–62. 10.1016/j.pharmthera.2015.11.012 26627987 PMC4747781

[B42] Ghyselinck J. Verstrepen L. Moens F. Van Den Abbeele P. Bruggeman A. Said J. (2021). Influence of probiotic bacteria on gut microbiota composition and gut wall function in an *in-vitro* model in patients with Parkinson’s disease. Int. J. Pharm. X 3, 100087. 10.1016/j.ijpx.2021.100087 34977556 PMC8683682

[B43] Gilbert J. Blaser M. J. Caporaso J. G. Jansson J. Lynch S. V. Knight R. (2018). Current understanding of the human microbiome. Nat. Med. 24, 392–400. 10.1038/nm.4517 29634682 PMC7043356

[B44] Guo C.-J. Allen B. M. Hiam K. J. Dodd D. Van Treuren W. Higginbottom S. (2019). Depletion of microbiome-derived molecules in the host using Clostridium genetics. Science 366, eaav1282. 10.1126/science.aav1282 31831639 PMC7141153

[B45] Guo T.-T. Zhang Z. Sun Y. Zhu R.-Y. Wang F.-X. Ma L.-J. (2023). Neuroprotective effects of sodium butyrate by restoring gut microbiota and inhibiting TLR4 signaling in mice with MPTP-induced Parkinson’s disease. Nutrients 15, 930. 10.3390/nu15040930 36839287 PMC9960062

[B46] Hall D. A. Voigt R. M. Cantu-Jungles T. M. Hamaker B. Engen P. A. Shaikh M. (2023). An open label, non-randomized study assessing a prebiotic fiber intervention in a small cohort of Parkinson’s disease participants. Nat. Commun. 14, 926. 10.1038/s41467-023-36497-x 36801916 PMC9938693

[B47] Hasegawa S. Goto S. Tsuji H. Okuno T. Asahara T. Nomoto K. (2015). Intestinal dysbiosis and lowered serum lipopolysaccharide-binding protein in Parkinson’s disease. PLOS ONE 10, e0142164. 10.1371/journal.pone.0142164 26539989 PMC4634857

[B48] Hawkes C. H. Del Tredici K. Braak H. (2007). Parkinson’s disease: a dual-hit hypothesis. Neuropathol. Appl. Neurobiol. 33, 599–614. 10.1111/j.1365-2990.2007.00874.x 17961138 PMC7194308

[B49] Hayes M. T. (2019). Parkinson’s disease and parkinsonism. Am. J. Med. 132, 802–807. 10.1016/j.amjmed.2019.03.001 30890425

[B50] Hegelmaier T. Lebbing M. Duscha A. Tomaske L. Tönges L. Holm J. B. (2020). Interventional influence of the intestinal microbiome through dietary intervention and bowel cleansing might improve motor symptoms in Parkinson’s disease. Cells 9, 376. 10.3390/cells9020376 32041265 PMC7072275

[B51] Heintz-Buschart A. Pandey U. Wicke T. Sixel-Döring F. Janzen A. Sittig-Wiegand E. (2018). The nasal and gut microbiome in Parkinson’s disease and idiopathic rapid eye movement sleep behavior disorder. Mov. Disord. 33, 88–98. 10.1002/mds.27105 28843021 PMC5811909

[B52] Hill-Burns E. M. Debelius J. W. Morton J. T. Wissemann W. T. Lewis M. R. Wallen Z. D. (2017). Parkinson’s disease and Parkinson’s disease medications have distinct signatures of the gut microbiome. Mov. Disord. 32, 739–749. 10.1002/mds.26942 28195358 PMC5469442

[B53] Holmqvist S. Chutna O. Bousset L. Aldrin-Kirk P. Li W. Björklund T. (2014). Direct evidence of Parkinson pathology spread from the gastrointestinal tract to the brain in rats. Acta Neuropathol. 128, 805–820. 10.1007/s00401-014-1343-6 25296989

[B54] Hopfner F. Künstner A. Müller S. H. Künzel S. Zeuner K. E. Margraf N. G. (2017). Gut microbiota in Parkinson disease in a northern German cohort. Brain Res. 1667, 41–45. 10.1016/j.brainres.2017.04.019 28506555

[B55] Huang H. Xu H. Luo Q. He J. Li M. Chen H. (2019). Fecal microbiota transplantation to treat Parkinson’s disease with constipation: a case report. Med. Baltim. 98, e16163. 10.1097/MD.0000000000016163 PMC6616439 31261545

[B56] Huang X.-Z. Li Z.-R. Zhu L.-B. Huang H.-Y. Hou L.-L. Lin J. (2014). Inhibition of p38 mitogen-activated protein kinase attenuates butyrate-induced intestinal barrier impairment in a Caco-2 cell monolayer model. J. Pediatr. Gastroenterol. Nutr. 59, 264–269. 10.1097/MPG.0000000000000369 24625969

[B57] Jackson A. Forsyth C. B. Shaikh M. Voigt R. M. Engen P. A. Ramirez V. (2019). Diet in Parkinson’s disease: critical role for the microbiome. Front. Neurol. 10, 1245. 10.3389/fneur.2019.01245 31920905 PMC6915094

[B58] Jankovic J. (2008). Parkinson’s disease: clinical features and diagnosis. J. Neurol. Neurosurg. Psychiatry 79, 368–376. 10.1136/jnnp.2007.131045 18344392

[B59] Kakoty V. K C S. Dubey S. K. Yang C.-H. Taliyan R. (2021). Neuroprotective effects of trehalose and sodium butyrate on preformed fibrillar form of α-synuclein-induced rat model of Parkinson’s disease. ACS Chem. Neurosci. 12, 2643–2660. 10.1021/acschemneuro.1c00144 34197084

[B60] Karunaratne T. B. Okereke C. Seamon M. Purohit S. Wakade C. Sharma A. (2020). Niacin and butyrate: nutraceuticals targeting dysbiosis and intestinal permeability in Parkinson’s disease. Nutrients 13, 28. 10.3390/nu13010028 33374784 PMC7824468

[B61] Keshavarzian A. Green S. J. Engen P. A. Voigt R. M. Naqib A. Forsyth C. B. (2015). Colonic bacterial composition in Parkinson’s disease. Mov. Disord. 30, 1351–1360. 10.1002/mds.26307 26179554

[B62] Kim T.-K. Bae E.-J. Jung B. C. Choi M. Shin S. J. Park S. J. (2022). Inflammation promotes synucleinopathy propagation. Exp. Mol. Med. 54, 2148–2161. 10.1038/s12276-022-00895-w 36473937 PMC9794777

[B63] Kouli A. Torsney K. M. Kuan W.-L. (2018). “Parkinson’s disease: etiology, neuropathology, and pathogenesis,” in Parkinson’s disease: pathogenesis and clinical aspects. Editors Stoker T. B. Greenland J. C. (Brisbane (AU): Codon Publications). 30702842

[B64] Kratsman N. Getselter D. Elliott E. (2016). Sodium butyrate attenuates social behavior deficits and modifies the transcription of inhibitory/excitatory genes in the frontal cortex of an autism model. Neuropharmacology 102, 136–145. 10.1016/j.neuropharm.2015.11.003 26577018

[B65] Lee H. M. Koh S.-B. (2015). Many faces of Parkinson’s disease: non-motor symptoms of Parkinson’s disease. J. Mov. Disord. 8, 92–97. 10.14802/jmd.15003 26090081 PMC4460545

[B66] Li C. Cui L. Yang Y. Miao J. Zhao X. Zhang J. (2019). Gut microbiota differs between Parkinson’s disease patients and healthy controls in northeast China. Front. Mol. Neurosci. 12, 171. 10.3389/fnmol.2019.00171 31354427 PMC6637281

[B67] Li T. Chu C. Yu L. Zhai Q. Wang S. Zhao J. (2022a). Neuroprotective effects of Bifidobacterium breve CCFM1067 in MPTP-induced mouse models of Parkinson’s disease. Nutrients 14, 4678. 10.3390/nu14214678 36364939 PMC9655354

[B68] Li W. Wu X. Hu X. Wang T. Liang S. Duan Y. (2017). Structural changes of gut microbiota in Parkinson’s disease and its correlation with clinical features. Sci. China Life Sci. 60, 1223–1233. 10.1007/s11427-016-9001-4 28536926

[B69] Li Z. Lu G. Luo E. Wu B. Li Z. Guo J. (2022b). Oral, nasal, and gut microbiota in Parkinson’s disease. Neuroscience 480, 65–78. 10.1016/j.neuroscience.2021.10.011 34695538

[B70] Lin A. Zheng W. He Y. Tang W. Wei X. He R. (2018). Gut microbiota in patients with Parkinson’s disease in southern China. Park. Relat. Disord. 53, 82–88. 10.1016/j.parkreldis.2018.05.007 29776865

[B71] Lin C.-H. Chen C.-C. Chiang H.-L. Liou J.-M. Chang C.-M. Lu T.-P. (2019). Altered gut microbiota and inflammatory cytokine responses in patients with Parkinson’s disease. J. Neuroinflammation 16, 129. 10.1186/s12974-019-1528-y 31248424 PMC6598278

[B72] Liu H. Wang J. He T. Becker S. Zhang G. Li D. (2018). Butyrate: a double-edged sword for health? Adv. Nutr. 9, 21–29. 10.1093/advances/nmx009 29438462 PMC6333934

[B73] Liu J. Wang F. Liu S. Du J. Hu X. Xiong J. (2017). Sodium butyrate exerts protective effect against Parkinson’s disease in mice via stimulation of glucagon like peptide-1. J. Neurol. Sci. 381, 176–181. 10.1016/j.jns.2017.08.3235 28991675

[B74] Louis P. Flint H. J. (2017). Formation of propionate and butyrate by the human colonic microbiota. Environ. Microbiol. 19, 29–41. 10.1111/1462-2920.13589 27928878

[B75] Lubomski M. Xu X. Holmes A. J. Muller S. Yang J. Y. H. Davis R. L. (2022). The gut microbiome in Parkinson’s disease: a longitudinal study of the impacts on disease progression and the use of device-assisted therapies. Front. Aging Neurosci. 14, 875261. 10.3389/fnagi.2022.875261 35656540 PMC9152137

[B76] Margolis K. G. Cryan J. F. Mayer E. A. (2021). The microbiota-gut-brain Axis: from motility to mood. Gastroenterology 160, 1486–1501. 10.1053/j.gastro.2020.10.066 33493503 PMC8634751

[B77] Markidi A. Elford J. D. Berkers C. Kraneveld A. D. Perez-Pardo P. (2024). “Chapter 9 - gut microbes in Parkinson’s disease: opportunities for microbial-based therapies,” in The gut-brain Axis. Editors Hyland N. Stanton C. Second Edition (United States: Academic Press), 217–240. 10.1016/B978-0-323-99971-7.00002-3

[B78] Martin-Gallausiaux C. Marinelli L. Blottière H. M. Larraufie P. Lapaque N. (2021). SCFA: mechanisms and functional importance in the gut. Proc. Nutr. Soc. 80, 37–49. 10.1017/S0029665120006916 32238208

[B79] Mazzoli R. Pessione E. (2016). The neuro-endocrinological role of microbial glutamate and GABA signaling. Front. Microbiol. 7, 1934. 10.3389/fmicb.2016.01934 27965654 PMC5127831

[B80] Melis M. Vascellari S. Santoru M. L. Oppo V. Fabbri M. Sarchioto M. (2021). Gut microbiota and metabolome distinctive features in Parkinson disease: focus on levodopa and levodopa-carbidopa intrajejunal gel. Eur. J. Neurol. 28, 1198–1209. 10.1111/ene.14644 33185912

[B81] Mertsalmi T. H. Aho V. T. E. Pereira P. a. B. Paulin L. Pekkonen E. Auvinen P. (2017). More than constipation - bowel symptoms in Parkinson’s disease and their connection to gut microbiota. Eur. J. Neurol. 24, 1375–1383. 10.1111/ene.13398 28891262

[B82] Minato T. Maeda T. Fujisawa Y. Tsuji H. Nomoto K. Ohno K. (2017). Progression of Parkinson’s disease is associated with gut dysbiosis: two-year follow-up study. PLoS One 12, e0187307. 10.1371/journal.pone.0187307 29091972 PMC5665539

[B83] Mitchell R. W. On N. H. Del Bigio M. R. Miller D. W. Hatch G. M. (2011). Fatty acid transport protein expression in human brain and potential role in fatty acid transport across human brain microvessel endothelial cells. J. Neurochem. 117, 735–746. 10.1111/j.1471-4159.2011.07245.x 21395585

[B84] Morrison D. J. Preston T. (2016). Formation of short chain fatty acids by the gut microbiota and their impact on human metabolism. Gut Microbes 7, 189–200. 10.1080/19490976.2015.1134082 26963409 PMC4939913

[B85] Murros K. E. Huynh V. A. Takala T. M. Saris P. E. J. (2021). Desulfovibrio bacteria are associated with Parkinson’s disease. Front. Cell Infect. Microbiol. 11, 652617. 10.3389/fcimb.2021.652617 34012926 PMC8126658

[B86] Nishiwaki H. Ito M. Hamaguchi T. Maeda T. Kashihara K. Tsuboi Y. (2022). Short chain fatty acids-producing and mucin-degrading intestinal bacteria predict the progression of early Parkinson’s disease. npj Park. Dis. 8, 65–12. 10.1038/s41531-022-00328-5 PMC9160257 35650236

[B87] Nuzum N. D. Loughman A. Szymlek-Gay E. A. Hendy A. Teo W.-P. Macpherson H. (2020). Gut microbiota differences between healthy older adults and individuals with Parkinson’s disease: a systematic review. Neurosci. Biobehav. Rev. 112, 227–241. 10.1016/j.neubiorev.2020.02.003 32032654

[B88] Nuzum N. D. Szymlek-Gay E. A. Loke S. Dawson S. L. Teo W.-P. Hendy A. M. (2023). Differences in the gut microbiome across typical ageing and in Parkinson’s disease. Neuropharmacology 235, 109566. 10.1016/j.neuropharm.2023.109566 37150399

[B89] Paiva I. Pinho R. Pavlou M. A. Hennion M. Wales P. Schütz A.-L. (2017). Sodium butyrate rescues dopaminergic cells from alpha-synuclein-induced transcriptional deregulation and DNA damage. Hum. Mol. Genet. 26, 2231–2246. 10.1093/hmg/ddx114 28369321

[B90] Pan S. Wei H. Yuan S. Kong Y. Yang H. Zhang Y. (2022). Probiotic Pediococcus pentosaceus ameliorates MPTP-induced oxidative stress via regulating the gut microbiota–gut–brain axis. Front. Cell Infect. Microbiol. 12, 1022879. 10.3389/fcimb.2022.1022879 36439235 PMC9682001

[B91] Pan-Montojo F. Schwarz M. Winkler C. Arnhold M. O’Sullivan G. A. Pal A. (2012). Environmental toxins trigger PD-like progression via increased alpha-synuclein release from enteric neurons in mice. Sci. Rep. 2, 898. 10.1038/srep00898 23205266 PMC3510466

[B92] Papić E. Rački V. Hero M. Tomić Z. Starčević-Čižmarević N. Kovanda A. (2022). The effects of microbiota abundance on symptom severity in Parkinson’s disease: a systematic review. Front. Aging Neurosci. 14, 1020172. 10.3389/fnagi.2022.1020172 36570528 PMC9772822

[B93] Pellegrini C. Antonioli L. Colucci R. Blandizzi C. Fornai M. (2018). Interplay among gut microbiota, intestinal mucosal barrier and enteric neuro-immune system: a common path to neurodegenerative diseases? Acta Neuropathol. 136, 345–361. 10.1007/s00401-018-1856-5 29797112

[B94] Perez-Pardo P. Broersen L. M. Kliest T. van Wijk N. Attali A. Garssen J. (2018). Additive effects of levodopa and a neurorestorative diet in a mouse model of Parkinson’s disease. Front. Aging Neurosci. 10, 237. 10.3389/fnagi.2018.00237 30127735 PMC6088190

[B95] Perez-Pardo P. de Jong E. M. Broersen L. M. van Wijk N. Attali A. Garssen J. (2017). Promising effects of neurorestorative diets on motor, cognitive, and gastrointestinal dysfunction after symptom development in a mouse model of Parkinson’s disease. Front. Aging Neurosci. 9, 57. 10.3389/fnagi.2017.00057 28373840 PMC5357625

[B96] Perez-Pardo P. Dodiya H. B. Engen P. A. Forsyth C. B. Huschens A. M. Shaikh M. (2019). Role of TLR4 in the gut-brain axis in Parkinson’s disease: a translational study from men to mice. Gut 68, 829–843. 10.1136/gutjnl-2018-316844 30554160

[B97] Petrov V. A. Saltykova I. V. Zhukova I. A. Alifirova V. M. Zhukova N. G. Dorofeeva Yu. B. (2017). Analysis of gut microbiota in patients with Parkinson’s disease. Bull. Exp. Biol. Med. 162, 734–737. 10.1007/s10517-017-3700-7 28429209

[B98] Pietrucci D. Cerroni R. Unida V. Farcomeni A. Pierantozzi M. Mercuri N. B. (2019). Dysbiosis of gut microbiota in a selected population of Parkinson’s patients. Park. Relat. Disord. 65, 124–130. 10.1016/j.parkreldis.2019.06.003 31174953

[B99] Plassais J. Gbikpi-Benissan G. Figarol M. Scheperjans F. Gorochov G. Derkinderen P. (2021). Gut microbiome alpha-diversity is not a marker of Parkinson’s disease and multiple sclerosis. Brain Commun. 3, fcab113. 10.1093/braincomms/fcab113 34704023 PMC8195527

[B100] Qian Y. Yang X. Xu S. Wu C. Song Y. Qin N. (2018). Alteration of the fecal microbiota in Chinese patients with Parkinson’s disease. Brain, Behav. Immun. 70, 194–202. 10.1016/j.bbi.2018.02.016 29501802

[B101] Qiao C.-M. Sun M.-F. Jia X.-B. Li Y. Zhang B.-P. Zhao L.-P. (2020). Sodium butyrate exacerbates Parkinson’s disease by aggravating neuroinflammation and colonic inflammation in MPTP-induced mice model. Neurochem. Res. 45, 2128–2142. 10.1007/s11064-020-03074-3 32556930

[B102] Ren T. Gao Y. Qiu Y. Jiang S. Zhang Q. Zhang J. (2020). Gut microbiota altered in mild cognitive impairment compared with normal cognition in sporadic Parkinson’s disease. Front. Neurol. 11, 137. 10.3389/fneur.2020.00137 32161568 PMC7052381

[B103] Rietdijk C. D. Perez-Pardo P. Garssen J. van Wezel R. J. A. Kraneveld A. D. (2017). Exploring braak’s hypothesis of Parkinson’s disease. Front. Neurol. 8, 37. 10.3389/fneur.2017.00037 28243222 PMC5304413

[B104] Rivière A. Selak M. Lantin D. Leroy F. De Vuyst L. (2016). Bifidobacteria and butyrate-producing colon bacteria: importance and strategies for their stimulation in the human gut. Front. Microbiol. 7, 979. 10.3389/fmicb.2016.00979 27446020 PMC4923077

[B105] Romano S. Savva G. M. Bedarf J. R. Charles I. G. Hildebrand F. Narbad A. (2021). Meta-analysis of the Parkinson’s disease gut microbiome suggests alterations linked to intestinal inflammation. npj Park. Dis. 7, 27–13. 10.1038/s41531-021-00156-z PMC7946946 33692356

[B106] Rosario D. Bidkhori G. Lee S. Bedarf J. Hildebrand F. Le Chatelier E. (2021). Systematic analysis of gut microbiome reveals the role of bacterial folate and homocysteine metabolism in Parkinson’s disease. Cell Rep. 34, 108807. 10.1016/j.celrep.2021.108807 33657381

[B107] Salvi P. S. Cowles R. A. (2021). Butyrate and the intestinal epithelium: modulation of proliferation and inflammation in homeostasis and disease. Cells 10, 1775. 10.3390/cells10071775 34359944 PMC8304699

[B108] Sampson T. R. Debelius J. W. Thron T. Janssen S. Shastri G. G. Ilhan Z. E. (2016). Gut microbiota regulate motor deficits and neuroinflammation in a model of Parkinson’s disease. Cell 167, 1469–1480. 10.1016/j.cell.2016.11.018 27912057 PMC5718049

[B109] Scheperjans F. Aho V. Pereira P. A. B. Koskinen K. Paulin L. Pekkonen E. (2015). Gut microbiota are related to Parkinson’s disease and clinical phenotype. Mov. Disord. 30, 350–358. 10.1002/mds.26069 25476529

[B110] Schmidt T. S. B. Raes J. Bork P. (2018). The human gut microbiome: from association to modulation. Cell 172, 1198–1215. 10.1016/j.cell.2018.02.044 29522742

[B111] Segal A. Zlotnik Y. Moyal-Atias K. Abuhasira R. Ifergane G. (2021). Fecal microbiota transplant as a potential treatment for Parkinson’s disease - a case series. Clin. Neurol. Neurosurg. 207, 106791. 10.1016/j.clineuro.2021.106791 34237681

[B112] Sharma S. Taliyan R. Singh S. (2015). Beneficial effects of sodium butyrate in 6-OHDA induced neurotoxicity and behavioral abnormalities: modulation of histone deacetylase activity. Behav. Brain Res. 291, 306–314. 10.1016/j.bbr.2015.05.052 26048426

[B113] Shin C. Lim Y. Lim H. Ahn T.-B. (2020). Plasma short-chain fatty acids in patients with Parkinson’s disease. Mov. Disord. 35, 1021–1027. 10.1002/mds.28016 32154946

[B114] Silva J. P. B. Navegantes-Lima K. C. Oliveira A. L. B. Rodrigues D. V. S. Gaspar S. L. F. Monteiro V. V. S. (2018). Protective mechanisms of butyrate on inflammatory bowel disease. Curr. Pharm. Des. 24, 4154–4166. 10.2174/1381612824666181001153605 30277149

[B115] Sivaprakasam S. Bhutia Y. D. Yang S. Ganapathy V. (2017). Short-chain fatty acid transporters: role in colonic homeostasis. Compr. Physiol. 8, 299–314. 10.1002/cphy.c170014 29357130 PMC6019286

[B116] Srivastav S. Neupane S. Bhurtel S. Katila N. Maharjan S. Choi H. (2019). Probiotics mixture increases butyrate, and subsequently rescues the nigral dopaminergic neurons from MPTP and rotenone-induced neurotoxicity. J. Nutr. Biochem. 69, 73–86. 10.1016/j.jnutbio.2019.03.021 31063918

[B117] St Laurent R. O’Brien L. M. Ahmad S. T. (2013). Sodium butyrate improves locomotor impairment and early mortality in a rotenone-induced Drosophila model of Parkinson’s disease. Neuroscience 246, 382–390. 10.1016/j.neuroscience.2013.04.037 23623990 PMC3721507

[B118] Sun M. F. Zhu Y. L. Zhou Z. L. Jia X. B. Xu Y. D. Yang Q. (2018). Neuroprotective effects of fecal microbiota transplantation on MPTP-induced Parkinson’s disease mice: gut microbiota, glial reaction and TLR4/TNF-α signaling pathway. Brain, Behav. Immun. 70, 48–60. 10.1016/j.bbi.2018.02.005 29471030

[B119] Svensson E. Horváth-Puhó E. Thomsen R. W. Djurhuus J. C. Pedersen L. Borghammer P. (2015). Vagotomy and subsequent risk of Parkinson’s disease. Ann. Neurology 78, 522–529. 10.1002/ana.24448 26031848

[B120] Takahashi K. Nishiwaki H. Ito M. Iwaoka K. Takahashi K. Suzuki Y. (2022). Altered gut microbiota in Parkinson’s disease patients with motor complications. Park. Relat. Disord. 95, 11–17. 10.1016/j.parkreldis.2021.12.012 34954497

[B121] Tamtaji O. R. Taghizadeh M. Kakhaki R. D. Kouchaki E. Bahmani F. Borzabadi S. (2019). Clinical and metabolic response to probiotic administration in people with Parkinson’s disease: a randomized, double-blind, placebo-controlled trial. Clin. Nutr. 38, 1031–1035. 10.1016/j.clnu.2018.05.018 29891223

[B122] Tan A. H. Chong C. W. Lim S.-Y. Yap I. K. S. Teh C. S. J. Loke M. F. (2021). Gut microbial ecosystem in Parkinson disease: new clinicobiological insights from multi-omics. Ann. Neurol. 89, 546–559. 10.1002/ana.25982 33274480

[B123] Tan A. H. Lim S. Y. Lang A. E. (2022). The microbiome-gut-brain axis in Parkinson disease - from basic research to the clinic. Nat. Rev. Neurol. 18, 476–495. 10.1038/s41582-022-00681-2 35750883

[B124] Turco L. Opallo N. Buommino E. De Caro C. Pirozzi C. Mattace Raso G. (2023). Zooming into gut dysbiosis in Parkinson’s disease: new insights from functional mapping. Int. J. Mol. Sci. 24, 9777. 10.3390/ijms24119777 37298727 PMC10253733

[B125] Turnbaugh P. J. Ridaura V. K. Faith J. J. Rey F. E. Knight R. Gordon J. I. (2009). The effect of diet on the human gut microbiome: a metagenomic analysis in humanized gnotobiotic mice. Sci. Transl. Med. 1, 6ra14. 10.1126/scitranslmed.3000322 PMC2894525 20368178

[B126] Unger M. M. Spiegel J. Dillmann K.-U. Grundmann D. Philippeit H. Bürmann J. (2016). Short chain fatty acids and gut microbiota differ between patients with Parkinson’s disease and age-matched controls. Park. Relat. Disord. 32, 66–72. 10.1016/j.parkreldis.2016.08.019 27591074

[B127] van Kessel S. P. de Jong H. R. Winkel S. L. van Leeuwen S. S. Nelemans S. A. Permentier H. (2020). Gut bacterial deamination of residual levodopa medication for Parkinson’s disease. BMC Biol. 18, 137. 10.1186/s12915-020-00876-3 33076930 PMC7574542

[B128] van Kessel S. P. Frye A. K. El-Gendy A. O. Castejon M. Keshavarzian A. van Dijk G. (2019). Gut bacterial tyrosine decarboxylases restrict levels of levodopa in the treatment of Parkinson’s disease. Nat. Commun. 10, 310. 10.1038/s41467-019-08294-y 30659181 PMC6338741

[B129] Van Laar T. Boertien J. M. Herranz A. H. (2019). Faecal transplantation, pro- and prebiotics in Parkinson’s disease; hope or hype? J. Parkinson’s Dis. 9, S371–S379. 10.3233/JPD-191802 31609702 PMC6839600

[B130] Vascellari S. Palmas V. Melis M. Pisanu S. Cusano R. Uva P. (2020). Gut microbiota and metabolome alterations associated with Parkinson’s disease. mSystems 5, e00561. 10.1128/mSystems.00561-20 32934117 PMC7498685

[B131] Vendrik K. E. Chernova V. O. Kuijper E. J. Terveer E. M. Hilten J. J. van Contarino M. F. (2023). Safety and feasibility of faecal microbiota transplantation for patients with Parkinson's disease: a protocol for a self-controlled interventional donor-FMT pilot study. BMJ Open 13, e071766. 10.1136/bmjopen-2023-071766 PMC10565159 37798034

[B132] Voigt R. M. Wang Z. Brown J. M. Engen P. A. Naqib A. Goetz C. G. (2022). Gut microbial metabolites in Parkinson’s disease: association with lifestyle, disease characteristics, and treatment status. Neurobiol. Dis. 170, 105780. 10.1016/j.nbd.2022.105780 35654277 PMC9241494

[B133] Walker A. W. Hoyles L. (2023). Human microbiome myths and misconceptions. Nat. Microbiol. 8, 1392–1396. 10.1038/s41564-023-01426-7 37524974

[B134] Wallen Z. D. Appah M. Dean M. N. Sesler C. L. Factor S. A. Molho E. (2020). Characterizing dysbiosis of gut microbiome in PD: evidence for overabundance of opportunistic pathogens. NPJ Park. Dis. 6, 11. 10.1038/s41531-020-0112-6 PMC7293233 32566740

[B135] Wallen Z. D. Demirkan A. Twa G. Cohen G. Dean M. N. Standaert D. G. (2022). Metagenomics of Parkinson’s disease implicates the gut microbiome in multiple disease mechanisms. Nat. Commun. 13, 6958. 10.1038/s41467-022-34667-x 36376318 PMC9663292

[B136] Wang H.-B. Wang P.-Y. Wang X. Wan Y.-L. Liu Y.-C. (2012). Butyrate enhances intestinal epithelial barrier function via up-regulation of tight junction protein Claudin-1 transcription. Dig. Dis. Sci. 57, 3126–3135. 10.1007/s10620-012-2259-4 22684624

[B137] Weis S. Schwiertz A. Unger M. M. Becker A. Faßbender K. Ratering S. (2019). Effect of Parkinson’s disease and related medications on the composition of the fecal bacterial microbiota. NPJ Park. Dis. 5, 28. 10.1038/s41531-019-0100-x PMC6884491 31815177

[B138] Wu G. Jiang Z. Pu Y. Chen S. Wang T. Wang Y. (2022). Serum short-chain fatty acids and its correlation with motor and non-motor symptoms in Parkinson’s disease patients. BMC Neurol. 22, 13. 10.1186/s12883-021-02544-7 34996385 PMC8740341

[B139] Xu R.-C. Miao W.-T. Xu J.-Y. Xu W.-X. Liu M.-R. Ding S.-T. (2022). Neuroprotective effects of sodium butyrate and monomethyl fumarate treatment through GPR109A modulation and intestinal barrier restoration on PD mice. Nutrients 14, 4163. 10.3390/nu14194163 36235813 PMC9571500

[B140] Yan H. Ajuwon K. M. (2017). Butyrate modifies intestinal barrier function in IPEC-J2 cells through a selective upregulation of tight junction proteins and activation of the Akt signaling pathway. PLoS One 12, e0179586. 10.1371/journal.pone.0179586 28654658 PMC5487041

[B141] Yang X. Ai P. He X. Mo C. Zhang Y. Xu S. (2022). Parkinson’s disease is associated with impaired gut-blood barrier for short-chain fatty acids. Mov. Disord. 37, 1634–1643. 10.1002/mds.29063 35607987

[B142] Zhang F. Yue L. Fang X. Wang G. Li C. Sun X. (2020). Altered gut microbiota in Parkinson’s disease patients/healthy spouses and its association with clinical features. Park. Relat. Disord. 81, 84–88. 10.1016/j.parkreldis.2020.10.034 33099131

[B143] Zhang Y. Xu S. Qian Y. He X. Mo C. Yang X. (2022). Sodium butyrate attenuates rotenone-induced toxicity by activation of autophagy through epigenetically regulating PGC-1α expression in PC12 cells. Brain Res. 1776, 147749. 10.1016/j.brainres.2021.147749 34896331

[B144] Zhang Y. Xu S. Qian Y. Mo C. Ai P. Yang X. (2023). Sodium butyrate ameliorates gut dysfunction and motor deficits in a mouse model of Parkinson’s disease by regulating gut microbiota. Front. Aging Neurosci. 15, 1099018. 10.3389/fnagi.2023.1099018 36761177 PMC9905700

[B145] Zhao Z. Ning J. Bao X. Shang M. Ma J. Li G. (2021). Fecal microbiota transplantation protects rotenone-induced Parkinson’s disease mice via suppressing inflammation mediated by the lipopolysaccharide-TLR4 signaling pathway through the microbiota-gut-brain axis. Microbiome 9, 226. 10.1186/s40168-021-01107-9 34784980 PMC8597301

[B146] Zhong Z. Ye M. Yan F. (2023). A review of studies on gut microbiota and levodopa metabolism. Front. Neurol. 14, 1046910. 10.3389/fneur.2023.1046910 37332996 PMC10272754

